# Effects of the Jigsaw method on student educational outcomes: systematic review and meta-analyses

**DOI:** 10.3389/fpsyg.2023.1216437

**Published:** 2023-08-03

**Authors:** Océane Cochon Drouet, Vanessa Lentillon-Kaestner, Nicolas Margas

**Affiliations:** ^1^Teaching and Research Unit in Physical Education and Sport, Haute Ecole Pédagogique du Canton de Vaud, Lausanne, Switzerland; ^2^Institute of Sport Sciences, University of Lausanne, Lausanne, Switzerland

**Keywords:** achievement, cooperative learning, learning, motivation, self-esteem, social relations

## Abstract

**Introduction:**

Cooperative learning methods are particularly interesting for building more inclusive schools; however, they have not been widely implemented. Among cooperative learning methods, the Jigsaw strategy is attractive for teachers, as it seems to be easy to implement and follow its four-step teaching structure; in addition, this method is believed to improve students' socialization and learning. To identify the effects of the Jigsaw method accurately, a systematic review of studies that have tested the effects of this method on important student educational outcomes was proposed and completed using a meta-analytical approach.

**Methods:**

A total of 69 Jigsaw studies were analyzed, and three major outcomes were retained following inductive and deductive thematic analyses: learning (including achievement and motivation), social relations, and self- esteem (including academic self-esteem and social self-esteem). When possible, complementary meta-analyses were conducted to quantify the Jigsaw effects on achievement (*n* = 43), motivation (*n* = 5), social relations (*n* = 4), and academic self-esteem (*n* = 4).

**Results:**

The primary results of our review focused on the inconsistency of Jigsaw effects and the high degree of variability among studies with regard to all retained student educational outcomes (i.e., achievement, motivation, social relations, and academic self-esteem) with the exception of social self-esteem, for which only three studies concluded that the Jigsaw method had positive effects. Moreover, homogeneous results were observed within studies. Our review highlights several factors that may explain this variability among studies: the sample size, the diversity of students in the classroom, and the type of content taught.

**Discussion:**

The moderating roles of these factors must be tested empirically, as they suggest ways of implementing the Jigsaw method more efficiently.

## 1. Introduction

Building more inclusive schools is a primary objective in international educational guidelines (UNESCO, 2009; ONU, 2015), and cooperative learning methods have been highlighted as one of the most effective ways to promote inclusion (Fabes et al., 2018; Farmer et al., 2019; Juvonen et al., 2019). Cooperative learning refers to a set of situations in which individuals interact and learn together (Johnson and Johnson, 1989; Slavin, 2011), and this form of learning has emerged as a promising pedagogical practice (Dyson and Casey, 2012). It permits students to engage collectively, viewing their peers as a fundamental learning resource. In this way, cooperative pedagogies offer interesting perspective on the improvement of both socialization and learning (for reviews, see Casey and Goodyear, 2015). There is a significant gap between the mass of scientific evidence in favor of cooperative learning and actual classroom practices. According to Pianta et al. (2007), in American schools, < 10% of class work takes the form of group work. This fact may be due to teachers' lack of confidence with regard to implementing this approach (Abrami et al., 2004), the complexity of cooperative learning principles (Buchs et al., 2017), a lack of training, ora concern about changing practices (Sabourin and Lehraus, 2008). Among cooperative learning methods, the Jigsaw method (Aronson et al., 1978) is an attractive method for teachers, as it proposes a four-step teaching structure that seems to be easy to follow. Jigsaw is thus one of the most popular and frequently studied cooperative learning methods. The purpose of the current paper is (1) to propose a systematic review of extant research on the Jigsaw method in education, (2) to identify the effects of the Jigsaw method on important student educational outcomes precisely and comprehensively, and (3) to highlight the limits of previous Jigsaw research and propose directions for future research and practical guidelines for the implementation of the Jigsaw method.

Originally developed by Aronson et al. (1978), the Jigsaw method aims to reduce intergroup prejudices in schools (Williams, 2004). After making some observations, Aronson et al. (1978) concluded that intergroup aggressiveness was due to the competitive classroom environment. Their idea was to create situations that would involve cooperative interracial interactions in which students would be dependent on one another to learn the material in a manner similar to assembling a jigsaw puzzle, with each member supplying an essential piece (Roseth et al., 2019). Thus, improving social relations among children was the first aim of the Jigsaw method. The Jigsaw method has evolved since 1978. Now, several models exist (see Table 1). Jigsaw I, which is the basis of each model, features four steps. (1) Students belong to a Jigsaw group. These groups exhibit within-group heterogeneity (i.e., sex, students' cognitive, social and motor levels) and between-group homogeneity and include 3–8 students each. (2) “Students join temporary “expert” groups consisting of students who have been assigned the same subset of material” (Roseth et al., 2019, p. 150). This step provides less competent students with the opportunity to learn how to understand and teach their material from more competent peers (Aronson and Patnoe, 2011; Roseth et al., 2019). (3) Students return to their original Jigsaw groups, where they are responsible for teaching and explaining the skills they have learned to their group members with the aim of making them competent as well. (4) Home group students work together to produce a final joint work through integration and evaluation.

**Table 1:** Historical evolution of the Jigsaw method.

**Version of the Jigsaw method**	**Creators**	**Characteristics**
Jigsaw I	Aronson et al. (1978)	Students alternate between working in the homegroup and the expert group; their roles and resources are complementary
Jigsaw II	Slavin (1986)	The structure is the same as that of Jigsaw I with the addition of a group reward based on the sum of individuals' performance in the group
Jigsaw III	Stahl (1994)	A quiz group is added before step 4 (correction)
Jigsaw IV	Holliday (2000)	A quiz and a test are added between each step
Subject Jigsaw	Doymus (2007)	This version is specific to the sciences (i.e., physics and chemistry); each student can see all the contents of the pre-expert step

Several meta-analyses have focused on the effects of cooperative learning on three important educational outcomes: achievement (Johnson et al., 2000; Kyndt et al., 2013; Stanczak, 2020), social relations and self-esteem (Slavin, 1990; Johnson et al., 2007; Kyndt et al., 2013). As cooperative learning can be defined in terms of situations in which teachers structure group work with the objective of maximizing social and cognitive gains (Buchs and Butera, 2015), these meta-analyses have included studies based on the Jigsaw method. First, as socialization is the primary objective of cooperative learning methods, Johnson et al. (2007) meta-analyzed the effects of cooperative learning on interpersonal relationships and self-esteem by examining 95 studies. These authors found that cooperative learning promotes greater liking among students than does competing with others [effect size (*ES)* = 0.68] or working on one's own in an individualistic manner (*ES* = 0.55) (Johnson et al., 2007). The results regarding self-esteem showed that cooperation promotes higher self-esteem than does competitive (*ES* = 0.47) or individualistic (*ES* = 0.29) behavior (Johnson et al., 2007). Nevertheless, numerous studies have been conducted since 2007, and this meta-analysis did not focus specifically on the Jigsaw method. Subsequently, Hattie (2017) produced a “mega-analysis” synthesis of the effects of cooperative learning on achievement and social relations, in which context the Jigsaw method was included among the top 10 most effective academic interventions, with an estimated ES of *d* = 1.20. This estimation is based on one meta-analysis of 11 studies conducted in Turkey between 2005 and 2012, with an average sample size of 109 participants (Stanczak et al., 2022). Such a large ES is unusual (Cheung and Slavin, 2016; Kraft, 2020; Patall, 2021) if we consider the first studies on the Jigsaw method (Johnson and Johnson, 2002) and the mean estimates in educational psychology (i.e., *d* = 0.33, see Schäfer and Schwarz, 2019). In addition, from this perspective on achievements pertaining to the Jigsaw method and cooperative learning, Slavin (2015) highlighted the importance of the motivational perspective. Nevertheless, to our knowledge, no review or meta-analysis has investigated the motivational effects of the Jigsaw method. Stanczak (2020) focused exclusively on the effects of the Jigsaw method. He conducted a meta-analysis of studies published between 2000 and 2019 that have tested the effects of the Jigsaw method on achievement in various academic domains. His results indicated a positive and large ES, *g* = 0.88, 95% CI [0.51; 1.25]; however, they were primarily characterized by a very large dispersion and significant heterogeneity, *Q*(df = 19) = 265.86, *p* < 0.001; I^2^ = 92.85%.

In summary, beyond achievement outcomes, other important outcomes, such as social relations, self-esteem, and motivation, must be investigated to identify the effects of the Jigsaw method more effectively and help practitioners make good decisions. Moreover, according to the theoretical relations among those outcomes, such a comprehensive approach can lead to a better understanding of the observed variability among studies pertaining to the effects of the Jigsaw method on achievement. This perspective also requires the identification of factors that could modify these Jigsaw effects.

To explain this variability in achievement more effectively and understand the effects of the Jigsaw method on important educational outcomes, several factors observed in the described methodologies were retained in this paper as potential moderators of the Jigsaw effects: the sample size, the duration of implementation, the discipline taught, the age of participants, and the diversity of students in the classroom. This choice relies on the possibility of observing these factors in the methodologies of the studies reviewed as well as theoretical and empirical considerations. First, the sample size is a potential determining factor. Indeed, ES estimates based on small samples are more sensitive to sampling error, which affects their precision and increases the likelihood of extreme estimates (Kühberger et al., 2014). Power analyses can be performed to estimate the number of participants required per experimental condition. These power calculations were performed by considering a minimum ES of interest of δ = 0.40 at a threshold of α = 0.05 (Maxwell, 2004; Stanczak et al., 2022), and the results indicated that 176 participants were needed to yield a 95% chance of detecting a medium effect (δ = 0.50), 140 participants for a 90% chance and 102 participants for an 80% chance. The duration of implementation is another factor that potentially influences the effect of the Jigsaw method. Indeed, efficient classroom implementation of the Jigsaw method requires preparation, adaptation and habituation. It takes time for students to familiarize themselves with the procedure (Aronson et al., 1978; Aronson and Patnoe, 2011; Roseth et al., 2019), and peer groups are more complex to establish and organize than individual procedures in which students work alone (e.g., Aydin and Biyikli, 2017; Roseth et al., 2019). Time allows the procedure to be routinized and potentially to be more effective. A recent study (Cochon Drouet et al., 2022) observed the influence of implementation time on the effects of the Jigsaw method. Concerning disciplines, some meta-analyses (Lou et al., 1996; Kyndt et al., 2013) have reported more positive effects of cooperative learning on “scientific” disciplines than others. However, in the meta-analysis conducted by Stanczak (2020) on the effect of the Jigsaw method on achievement, no significant differences between studies conducted in one scientific discipline and those conducted in another discipline were detected. It is possible to explore the teaching content using the Jigsaw method, as it breaks down the teaching objectives into subcategories on which the expert groups can work (Aronson and Patnoe, 2011). Student age can be a potential factor. Indeed, Kyndt et al. (2013) observed differences across students' ages, with higher effects of cooperative learning being found at the primary and tertiary levels than at the secondary level. However, no explanation of the origin of these differences was given. Finally, student diversity (i.e., student heterogeneity in terms of achievement or sex) is also a potential factor. Cohen (1994) explained that diversity can be problematic for cooperation in class. Excessive diversity can be detrimental to mutual understanding, but it can also be a source of progress and social cohesion, with diversity being viewed as an asset (Peyrat, 2009).

To legitimate the Jigsaw method in actual and future pedagogical practices, we must go beyond the intuitive notion that the method should work and identify its effects more precisely. A previous study reported promising results regarding specific achievement outcomes (e.g., Stanczak, 2020), but practical decisions must consider all educational outcomes of the Jigsaw method from a comprehensive perspective. Moreover, the large dispersion and significant heterogeneity among studies on students' achievement observed by Stanczak (2020) must be analyzed in more depth. We must evaluate whether this variability can also be found in other educational outcomes and identify the potential factors that could explain such hypothetical variability among studies. These factors leading to the differential impacts of Jigsaw on important educational outcomes are also crucial to improve the Jigsaw method. Therefore, the purpose of the current paper was (1) to propose a review of the extant research on the Jigsaw method in education, (2) to identify the effects of the method on each student's important educational outcomes, and (3) to improve our understanding of the heterogeneity of these effects with the aim of proposing directions for future research and guidelines regarding the implementation of the Jigsaw method. In other words, two research questions were raised in this study:

1. What effects does the jigsaw method have on different educational outcomes?
2. Do the results regarding different outcomes exhibit heterogeneity? If so, why?

To achieve these goals, a systematic review was conducted to identify the educational outcomes empirically associated with the Jigsaw method. When possible, we proceeded to perform a complementary meta-analysis of each identified outcome variable with the aim of quantifying the observed Jigsaw effects and their variability across studies. Finally, we tried to identify the factors that could explain such potential variability. Based on previous research and theoretical propositions, our examination of the literature focused more deeply on the five following factors: the sample size, the discipline taught, the age of the students, the duration of implementation, and the diversity among the students.

## 2. Methods

The systematic review and meta-analyses followed the Preferred Reporting Items for Systematic Reviews and Meta-Analyses (PRISMA) guidelines for reporting meta-analytical findings (Moher et al., 2009). The identification of relevant studies was conducted by reference to the following databases: *ERIC, ScienceDirect, Taylor and Francis, Web of Science, Cairn*, and *APA Psychnet*. The keyword “Jigsaw” was used, and the combination of the following keywords was used with the Boolean connector “AND”: Jigsaw AND Education. The whole process was implemented in April 2023.

### 2.1. Systematic review

Only published or accepted peer-reviewed scientific education research articles that were written in English or French, contained the keywords “Jigsaw” and “Education” in the body of the article, included information about the research goal (excluding theoretical studies and reviews) and were published in an education journal referenced by Scimago Journal and Country Rank (SJR) were considered for the review. Abstracts and conference papers were not included.

**Figure 1:**
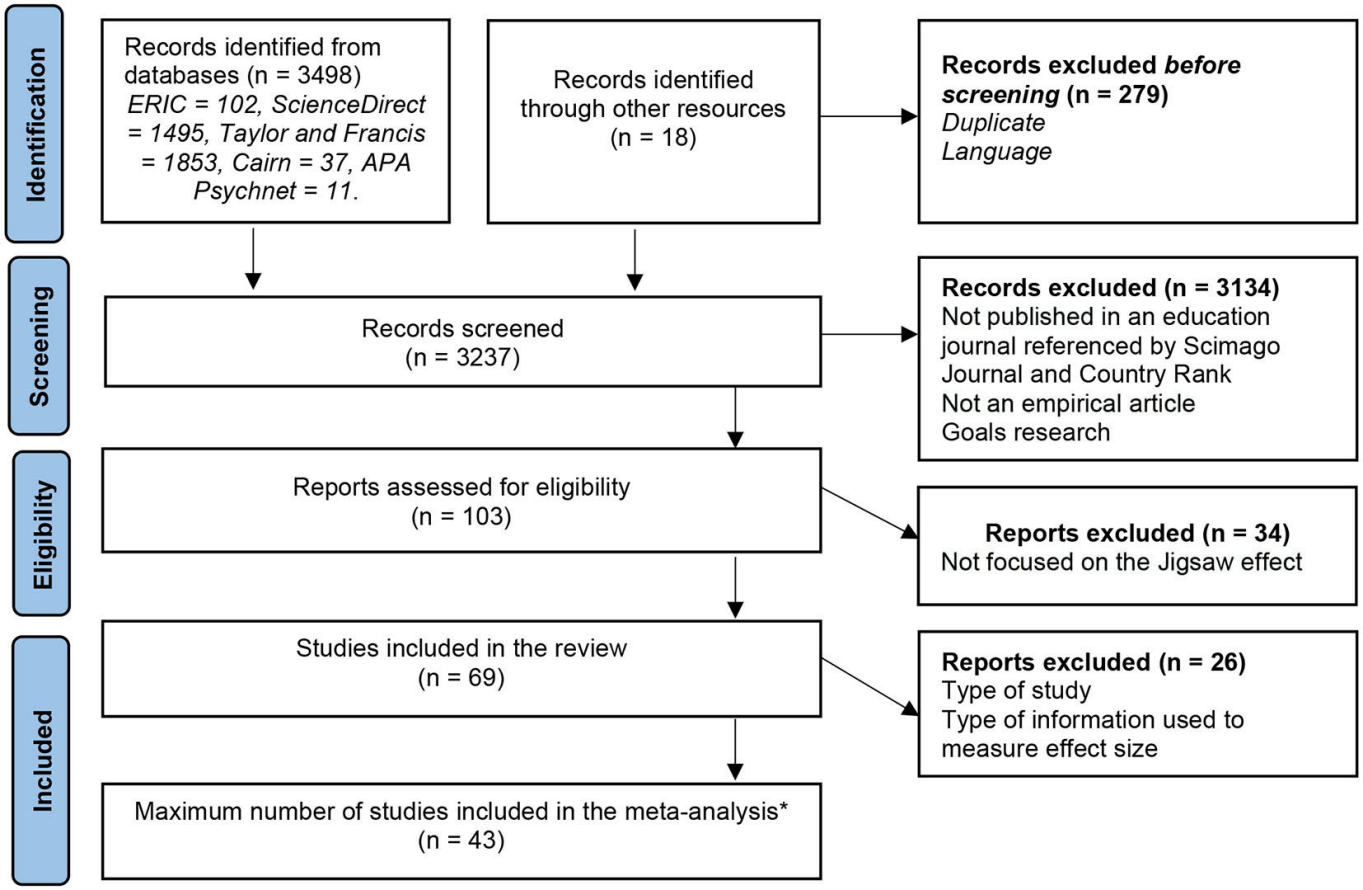
Prisma flowchart of the identification process of the systematic review and meta-analysis. ^*^Only the achievement meta-analysis included the maximum of the studies retained (*n* = 43).

Figure 1 shows the flow diagram for the literature selection process. The full screening process followed the Prisma guidelines (Page et al., 2021). The first step of the review consisted of checking all retained references (*n* = 3,237) to eliminate duplicates. In the second step, the titles and abstracts of the selected articles were examined to determine applicability, and the full texts were read carefully to determine whether they conformed to the inclusion criteria. The sample was thus reduced to 103 references. In the third step, as recommended by Scott et al. (2018), we verified the quality of these 103 studies. The studies included in this review were checked by the first and second authors. Two criteria were consulted. First, the study in question should present a well-defined argument that establishes a connection between theory and research, showcasing a logical and coherent line of reasoning. It should effectively elucidate the relevant theoretical foundations and previous research, contributing to the formulation of the research question(s). Second, the study should present its findings and makes claims that align with and are supported by the methods used in the research.

Consensus was reached between the two coders to delete 34 studies from this set of 103. Ultimately, 69 studies were included in this review. The variables used in the coding procedure are detailed in Table 2.

**Table 2:** Description of the studies retained in this review.

**Date of publication (year)**	***N***	**Percent**
1975–1980	2	2.90
1980–1985	3	4.35
1985–1990	1	1.45
1990–1995	2	2.90
1995–2000	1	1.45
2000–2005	1	1.45
2005–2010	19	27.54
2010–2015	12	17.39
After 2015	28	40.58
**Sample size (n)**		
>176	12	17.39
140–175	3	4.35
102–139	10	14.49
50–101	32	43.38
< 50	13	18.84
**Geographic location**		
East	42	60.87
West	27	39.13
**Grade level**		
Primary school	16	23.19
Secondary school	20	28.99
University	33	47.82
**Jigsaw method**		
Jigsaw I	51	82.61
Jigsaw II	8	11.59
Jigsaw III	1	1.45
Jigsaw IV	3	4.35
Subject Jigsaw	6	8.69
**Duration of implementation**		
> 15 h	18	26.09
10–15 h	11	15.94
9–5 h	20	28.99
< 5 h	16	23.19
Not mentioned	4	5.80
**Discipline**		
Sciences	37	53.62
Languages	9	13.04
Social sciences	10	14.49
Physical education	5	7.25
Not mentioned	8	11.59

Subsequently, these 69 studies were analyzed using a two-step process. First, the studies were categorized based on the following parameters: year of publication, sample size, academic level of students, discipline, geographic location, and duration of implementation. Second, a more in-depth content analysis of the studies' methods and results facilitated the identification of several themes and subthemes. Both inductive and deductive thematic analyses were used as described by Fereday and Muir-Cochrane (2006). The identification of themes and grouping into major themes were performed by two researchers, and the results were compared until consensus was reached. Finally, three major educational outcomes and certain subthemes were identified by reference to the extant research on some of the effects of the Jigsaw method: learning (encompassing motivation and achievement), social relations, and self-esteem (including academic self-esteem and social self-esteem).

### 2.2. The meta-analyses

The first step of this meta-analytical approach consisted of checking all statistical information contained in the 69 studies included in the systematic review. Studies that met all the following inclusion criteria were selected for the meta-analyses. Only empirical studies were selected. Such studies were required to include both a control group and an experimental group. Only studies that contained sufficient information (N, M, SD) to measure the computation of effect size were included. Studies featuring only surveys, studies featuring only a one-group pretest and posttest design, and qualitative studies were excluded.

The 69 studies were analyzed by the first and second authors. Consensus was reached between the two coders to delete 26 studies from this set of 69 because they did not meet all the inclusion criteria.

The same themes that had previously been identified in the systematic review were used, and regarding the theme “students' learning”, two meta-analyses were performed – one meta-analysis on achievement and one on motivation. The “social relations” theme was the focus of one meta-analysis. “Self-esteem” was explored through one meta-analysis on academic self-esteem. The specific constructs of the variables are specified in the Appendix. When a meta-analysis could be conducted, each educational outcome was studied using a mixed-methods approach combining a qualitative analysis of the results and a meta-analysis of the obtained effect sizes.

Several elements were included in the data analysis. The mean and standard deviation were extracted to estimate the average ES and its dispersion using the *g* parameter developed by Hedge (Borenstein et al., 2009). First, a Cohen *d*, i.e., the standardized difference between the means of the two experimental groups (Jigsaw vs. Control) as weighted by an intrastudy standard deviation, was calculated for each study. Then, this value was corrected alongside its associated variance by a “small-sample correction” factor *J* to arrive at the Hedge parameter *g*, which can be interpreted using the benchmarks defined by Hattie (2009). Hattie (2009) defined a small ES as δ = 0.20; a medium ES as δ = 0.40, which was motivated by the criteria of practice signification and his estimation of a medium ES of cooperative learning of δ = 0.41; and a large ES as δ = 0.8. If a study reported multiple measures of the same construct, an intermeasurement correlation coefficient *r* = 0.71 was estimated based on the recommendations of Borenstein et al. (2009). This coefficient permits us to measure a variance inflation factor (VIF), which is used to correct the inflation of the variance in studies that used many measures of the same proxy. We used the “MAJOR” extension to conduct our meta-analysis with the support of Jamovi 1.1.8.0 software. As Goh et al. (2016) noted, this approach is usually conservative if few studies are available, but it offers greater generalizability. The Der-Simonian-Laird method was used for the computation of the estimate because it is one of the most frequently used approaches and is simple to implement (Veroniki et al., 2016). We estimated the homogeneity of effects using Q-statistics and I^2^ (Higgins et al., 2003), which relate the observed variance to the within-study variance, with the goal of highlighting potential sources of heterogeneity.

Publication bias was examined using funnel plot asymmetry (Tatsioni and Ioannidis, 2017). This approach focuses on a plot of the estimate of ES in each study against an estimate of its precision (typically its standard error; Simmonds, 2015). In a funnel plot, smaller studies tend to exhibit larger variation in their estimated treatment effects, resulting in more scattered data points around the mean effect. In contrast, larger studies exhibit smaller variation and cluster more closely around the mean effect (Simmonds, 2015). This pattern arises due to the inherent uncertainty associated with smaller sample sizes, which leads to a wider range of estimated effects (Tatsioni and Ioannidis, 2017). Moreover, Tables 3–6 show the role of sample size in the change in ESs (Uttl et al., 2017).

**Table 3:** Estimated effect sizes and characteristics of students' achievement.

**Study**	**Effect size**	**Confidence intervals**	**Sample**	**Study weight**	**Population**	**Discipline**	**Duration (hours)**	**Percent boys**
Göçer (2010)	4.29	[3.88, 4.70]	60	1.90	SS	Literature	3	NM
Yapici (2016)	2.65	[2.38, 2.92]	53	2.12	SS	Sciences	11	NM
Tarhan et al. (2013)	2.57	[2.33, 2.81]	61	2.15	SS	Physics	8	NM
Gömleksi'z (2007)	2.38	[2.09, 2.67]	66	2.09	UNI	English	28	NM
Koc et al. (2010)	1.90	[1.76, 2.04]	106	2.39	UNI	Chemistry	16	NM
Doymus (2008b)	1.75	[1.36, 2.14]	68	1.93	UNI	Chemistry	10	NM
Abed et al. (2020)	1.49	[1.36, 1.61]	80	2.43	SS	Mathematics	8	0
Doymus et al. (2010)	1.47	[1.33, 1.61]	122	2.39	UNI	Chemistry	5	NM
Tarhan and Sesen (2012)	1.39	[1.13, 1.64]	38	2.19	UNI	Chemistry	1.15	NM
Aydin and Biyikli (2017)	1.31	[1.17, 1.45]	63	2.39	UNI	Physics	12	NM
Akkus and Doymuş (2022)	1.18	[0.56,1.80]	68	2.27	PS	Sciences	NM	52.70
Er (2017)	1.14	[0.85, 1.43]	46	2.09	SS	Social sciences	12	58.70
Kilic (2008)	1.13	[0.69,1.57]	80	2.48	UNI	Pedagogy	NM	NM
Artut and Tarim (2007)	1.09	[0.97, 1.21]	71	2.43	UNI	Mathematics	36	NM
Karacop and Doymus (2013)	1.09	[0.91, 1.27]	115	2.31	UNI	Chemistry	5	44.40
Namaziandost and Gilakjani (2020)	1.09	[0.89, 1.25]	50	2.31	SS	English	20	100
Doymus (2008a)	1.04	[0.88, 1.20]	36	2.35	UNI	Chemistry	9	NM
Sahin (2010)	0.97	[0.87, 1.07]	80	2.48	UNI	Turkish	24	NM
Doymus (2007)	0.96	[0.86, 1.06]	108	2.48	UNI	Chemistry	12	NM
Sahin (2011)	0.86	[0.74, 0.98]	71	2.43	PS	Writing	24	45.00
Hornby (2009)	0.76	[0.58, 0.94]	44	2.31	UNI	Education	2	NM
Van Dat (2016)	0.54	[0.10, 0.98]	80	2.48	UNI	Management	18	60.00
Koç et al. (2016)	0.53	[0.35, 0.71]	71	2.31	SS	Sciences	20	6.8
Wilson et al. (2017)	0.46	[0.36, 0.56]	94	2.48	UNI	Pharmaceutic	12	38.30
Garcia (2021)	0.45	[0.01, 0.89]	80	2.48	UNI	Informatics	14	93.75
Arslan (2016)	0.38	[0.24, 0.52]	56	2.75	SS	Turkish	12	51.80
Sagsoz et al. (2017)	0.34	[−0.18, 0.86]	50	2.39	UNI	Dentistry	3	NM
Ghaith and El-Malak (2004)	0.27	[0.09, 0.45]	48	2.31	SS	Reading	15	60.40
Roseth et al. (2019)	0.26	[0.24, 0.28]	258	2.68	UNI	Anatomy	10	32.00
Shaaban (2006)	0.23	[−0.02, 0.48]	45	2.15	SS	Reading	8	59.10
Suárez-Cunqueiro et al. (2017)	0.21	[0.13, 0.29]	109	2.53	UNI	Dentistry	12	31.20
Costouros (2020)	0.18	[−0.21, 0.57]	50	2.53	UNI	Management	48	54.00
Oakes et al. (2019)	0.15	[−0.87, 0.36]	145	2.31	UNI	Anatomy	2	23.40
Stanczak et al. (2022) study 4	0.05	[−0.18, 0.27]	74	2.48	SS	Earth and life sciences	18	48.65
Stanczak et al. (2022) study 5	0.05	[−0.14, 0.25]	101	2.53	SS	Earth and life sciences	18	43.56
Ural et al. (2017)	0.01	[−0.15, 0.17]	49	2.35	PS	Sciences	6	NM
Stanczak et al. (2022) study 2	0	[−0.22, 0.22]	313	2.68	SS	Earth and life sciences	2	46.00
Stanczak et al. (2022) study 1	−0.04	[−0.30, 0.2]	252	2.63	SS	Mathematics	2	41.66
Stanczak et al. (2022) study 3	−0.07	[−0.26, 0.11]	110	2.58	SS	Physics and chemistry	16	52.73
Berger and Hänze (2009)	−0.07	[−0.11, −0.03]	344	2.63	SS	Physics	9	67.00
Hänze and Berger (2007)	−0.24	[−0.30, −0.18]	137	2.58	SS	Physics	9	NM
Souvignier and Kronenberger (2007)	−0.48	[−0.52, −0.44]	208	2.63	PS	Sciences	15	48.10
Moreno (2009)	−0.65	[−0.79, −0.51]	87	2.39	UNI	Biology	1	63.10
*K* = 42	0.77	[0.55, 0.98]		100				

NM, Not mentioned; PS, Primary school; SS, Secondary school; UNI, University.

For each selected outcome, we tried to understand the heterogeneity of the results observed in both the systematic review and meta-analysis more accurately. Moderation analyses seek to test whether study groupings explain differences in heterogeneity, i.e., differences in the dispersion of Jigsaw effects (Stanczak, 2020). Five potential moderators were analyzed in depth to try to explain this heterogeneity (e.g., the sample size, the discipline taught, the age of the students, the duration of implementation, and the diversity among the students). This approach enables us to compare several studies according to a specific criterion. For reasons of statistical power and the categorical nature of some moderators, we chose to divide each moderator into two categories. In the case of continuous variables, categorical classification was performed based on the median score of our sample (Stanczak, 2020). More precisely, concerning the duration of implementation, in our sample, we used the median duration in hours (i.e., Δt = 9) to categorize “short” or “long” durations of implementation and test the moderating effect by reference to Jigsaw exposure duration. Concerning the sample size, the 102 participant range was chosen according to the power of the studies. Concerning the discipline taught, the sciences were separated from other disciplines. Concerning the students' age, schoolchildren were separated from students in higher education. Finally, no quantitative exploration of moderation was performed with regard to students' diversity due to overly heterogeneous criteria.

## 3. Results

This section presents the results first of the systematic review, second of the meta-analyses, and third regarding the factors that could explain the heterogeneity in results.

### 3.1. Systematic review

Table 2 shows a general description of the selected studies, and the Appendix provides a complete description of each selected study. The number of studies increased over the period (1976–2022), and the majority of studies were published after 2000 (85.50%). The focus of these studies ranged from primary schools to universities, and 60% of the students were conducted in an Eastern context (see Table 2). The mean duration of the implementation of the Jigsaw method was 13.76 hours. The results of the systematic review of each outcome are detailed below.

#### 3.1.1. Students' perceptions of achievement and learning

Among the 48 studies on students' achievement, 18 focused on students' perceptions of achievement and learning with respect to the Jigsaw method (e.g., Aydin and Biyikli, 2017; Er, 2017). Seven studies involved interviews, and 13 entailed open-ended items in questionnaires. Some studies showed improvements in perceived achievement or learning. For example, in the study conducted by Aydin and Biyikli (2017), 33% of students reported that tasks were perceived easier in the Jigsaw condition. The interviews conducted by Tarhan et al. (2013) revealed that “83% of students thought that since they were responsible for their own learning (i.e., mastery climate), they were meeting the lesson's objectives” (p. 196). These qualitative results support the positive effect of the Jigsaw method on learning.

#### 3.1.2. Students' motivation

Six studies measured intrinsic and extrinsic motivation in scientific disciplines (Hänze and Berger, 2007; Berger and Hänze, 2009, 2015; Roseth et al., 2019; Sanaie et al., 2019; Costouros, 2020). Their results are detailed in the meta-analysis section. Three other studies used other motivational constructs (Ural et al., 2017; Blajvaz et al., 2022; Cochon Drouet et al., 2022). For example, Ural et al. (2017) showed a positive Jigsaw effect on motivation to learn (*g* = 0.6), and Cochon Drouet et al. (2022) obtained mixed results regarding physical education, including significant positive or negative effects of the Jigsaw method on situational interest and motor engagement according to the type of sport taught in the context of physical education as compared to the control condition. Berger and Hänze (2015) also showed that the type of content taught using the Jigsaw method in the context of physics modified teachers' quality of teaching and their intrinsic motivation. The more demanding the content is, the higher the cognitive load faced by the student, and the more this factor hinders motivation.

#### 3.1.3. Students' social relations

In terms of student social relations, the Jigsaw effect appeared to be inconsistent. Indeed, some studies obtained positive results, others obtained negative results, and one yielded mixed results. More precisely, positive effects of the Jigsaw method on social relations were found in 11 studies (Blaney et al., 1977; Bridgeman, 1981; Ziegler, 1981; Desforges et al., 1991; Lazarowitz et al., 1994; Walker and Crogan, 1998; Hänze and Berger, 2007; Göçer, 2010; Theobald et al., 2017; Oakes et al., 2019; Costouros, 2020; Chang and Benson, 2022). For example, in Desforges et al. (1991 study), the Jigsaw method led to more positive interpersonal attitudes and more empathy. The study conducted by Theobald et al. (2017) found positive effects on students' social relations.

The study conducted by O'Leary et al. (2019) presented mixed results with regard to social relations depending on students' ability level. Indeed, in this study, which focused on the context of physical education, some students recognized that high-ability classmates could help their lower-ability peers learn (O'Leary et al., 2019). However, the results also showed that the low ability of students negatively impacted their social relationships. For example, one student in this study stated that “two of us worked truly well, and the other one (a low practical ability student), I truly struggled to connect with. I was trying to determine where he was at and where he felt comfortable and to get him more involved” (O'Leary et al., 2019, p. 723).

Finally, ten studies concluded that the effect of the Jigsaw method on social relations was either inconsistent (Moskowitz et al., 1983, 1985; Santos Rego and Moledo, 2005; Berger and Hänze, 2009; Zacharia et al., 2011; Roseth et al., 2019) or negative (Bratt, 2008; O'Leary and Griggs, 2010; O'Leary et al., 2015; Aydin and Biyikli, 2017). Indeed, the study conducted by O'Leary et al. (2015) identified problems resulting from group heterogeneity. These authors observed that it was difficult for many students to teach their peers. For example, one participant noted that “students seem to lack basic skills to talk and listen to each other; teaching each other becomes almost impossible” (O'Leary et al., 2015, p. 186). Overall, 7% of students in the study conducted by Aydin and Biyikli (2017) reported conflicts within groups in which the lack of knowledge on the part of one group member negatively affected other group members. The results of the study conducted by Roseth et al. (2019) in the context of anatomy also suggested that “increasing epistemic regulation may have the unintended effect of also making social comparison more salient, as deeper engagement with the material also heightens students' sensitivity to relative differences in competence” (p. 162).

#### 3.1.4. Students' social self-esteem

Three studies focused only on social self-esteem (Blaney et al., 1977; Lazarowitz et al., 1994; Walker and Crogan, 1998). Two studies showed a significant increase in social self-esteem in favor of the Jigsaw condition (Blaney et al., 1977; Lazarowitz et al., 1994), and one study reported nonsignificant results (Walker and Crogan, 1998). The lack of statistical information prevented us from conducting a meta-analysis on this topic.

#### 3.1.5. Students' academic self-esteem

A positive effect of the Jigsaw method on academic self-esteem was found in two studies (Hänze and Berger, 2007; Crone and Portillo, 2013), and a nonsignificant effect was found in the four other studies (Moskowitz et al., 1983; Berger and Hänze, 2009; Roseth et al., 2019; Costouros, 2020). In the study conducted by Crone and Portillo (2013), undergraduate students had more confidence in their ability to communicate orally about psychology. The results reported by Hänze and Berger (2007) in the context of physics in secondary school showed that students with a low academic self-concept had a greater feeling of competence in the Jigsaw condition than in the direct instruction condition. Moreover, the results showed a significant interaction of the Jigsaw condition with gender, resulting in a greater feeling of competence for girls than in the traditional teaching setting in the context of physics, which is viewed as a masculine discipline (Morge and Toczek, 2009). Three studies reported no significant results.

### 3.2. Meta-analysis

#### 3.2.1. Student achievement

**Figure 2:**
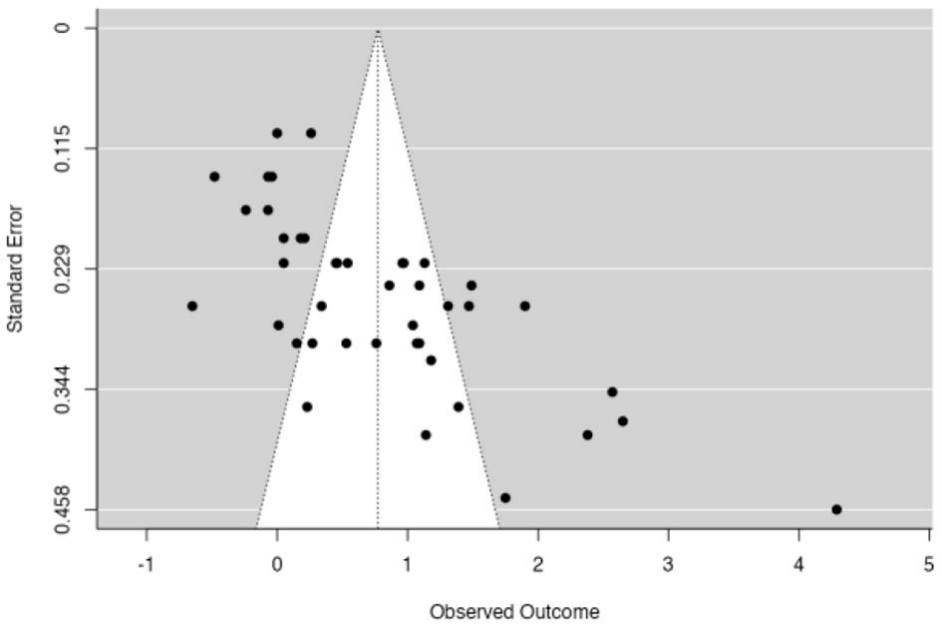
Funnel plot of the effect sizes of achievement outcomes. The outer dashed lines indicate the triangular region within which 95% of studies are expected to be located in the absence of both biases and heterogeneity (fixed effect summary log odds ratio ± 1.96 × standard error of summary log odds ratio). The solid vertical line corresponds to no intervention effect (Sterne et al., 2011). The large samples are on the left, and the small samples are on the right.

Our meta-analysis of student achievement included 43 studies (five experiments conducted by Stanczak et al., 2022 and 38 articles on student achievement that were included in a meta-analysis, see Table 3 and Figure 2). It revealed a large ES of the Jigsaw method on achievement (*g* = 0.77, 95% CI [0.55; 0.98], *p* < 0.001). However, this meta-analysis also showed a large dispersion and significant heterogeneity [*Q*(df=41) = 457.04, *p* < 0.001, τ^2^ = 0.48, *I*^2^ = 91.03]. Some studies (Hänze and Berger, 2007; Souvignier and Kronenberger, 2007; Moreno, 2009; 9.30% of the 43 studies, Berger and Hänze, 2009), including three with large sample sizes, even indicated significant negative ES (*g* = [−0.65; −0.07]). Among the 43 studies on student achievement included in the meta-analysis, 17 had an ES > 1 that could be of substantial concern (Cheung and Slavin, 2016). The mean sample size was 69 participants (the mean sample size of all studies in our meta-analysis was 94 participants). Among these 17 studies, 12 were conducted at universities with a focus on the sciences, and 16 were conducted in Turkey.

Figure 2 offers a visual representation of heterogeneity (i.e., publication bias).

#### 3.2.2. Student motivation

**Figure 3:**
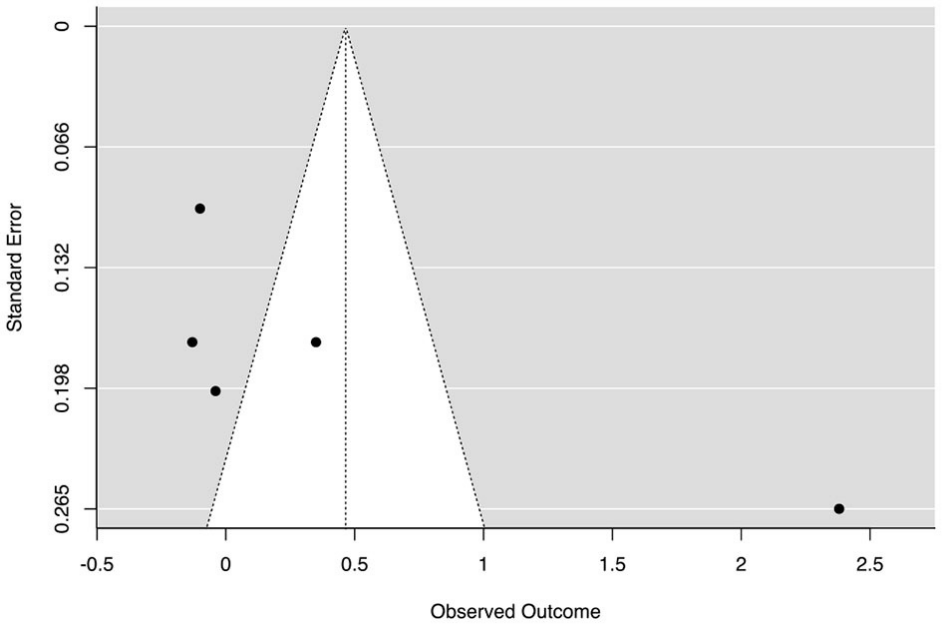
Funnel plot of the effect sizes of motivation outcomes. The outer dashed lines indicate the triangular region within which 95% of studies are expected to be located in the absence of both biases and heterogeneity (fixed effect summary log odds ratio ± 1.96 × standard error of summary log odds ratio). The solid vertical line corresponds to no intervention effect (Sterne et al., 2011). The large samples are on the left, and the small samples are on the right.

With regard to the five studies that measured intrinsic and extrinsic motivation in scientific disciplines (Hänze and Berger, 2007; Berger and Hänze, 2009, 2015; Roseth et al., 2019; Sanaie et al., 2019; Costouros, 2020), a meta-analysis was possible, as Berger and Hänze (2015) used a design without control conditions. This meta-analysis revealed non-significant ESs (*g* = 0.47, 95% CI [−0.21, 1.13], *p* = 0.17; see Table 4 and Figure 3) and significant heterogeneity among these studies [*Q*(df = 5) = 82.14, *p* < 0.001, τ^2^ = 0.55, *I*^2^ = 95.1].

**Table 4:** Estimated effect size and characteristics of students' intrinsic and extrinsic motivation.

**Study**	**Effect size**	**Confidence intervals**	**Sample**	**Study weight**	**Population**	**Discipline**	**Duration (hours)**
Sanaie et al. (2019)	2.38	[2.24, 2.52]	94	18.88	UNI	Nursing	34
Hänze and Berger (2007)	0.35	[0.29, 0.41]	137	20.19	SS	Physics	9
Costouros (2020)	−0.04	[−0.43, 0.35]	50	19.84	UNI	Management	48
Roseth et al. (2019)	−0.10	[−0.12, −0.08]	258	20.91	UNI	Anatomy	10
Berger and Hänze (2009)	−0.13	[−0.19, −0.07]	286	20.19	SS	Physics	9
*K* = 4	0.46	[−0.20, 1.13]		100			

SS, Secondary school; UNI, University.

Figure 3 offers a visual representation of heterogeneity (i.e., publication bias).

#### 3.2.3. Student social relations

**Figure 4:**
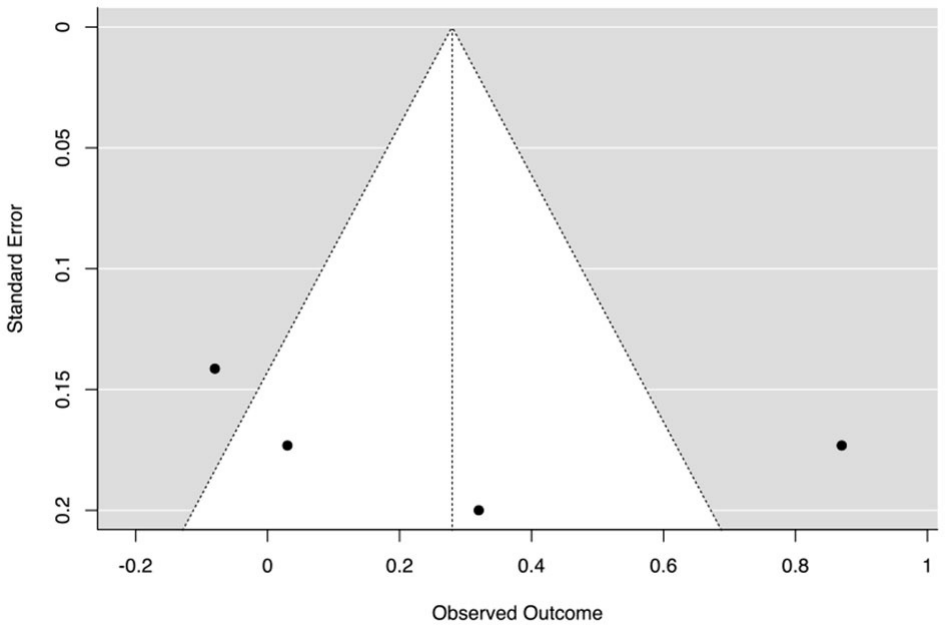
Funnel plot of the effect sizes of relatedness outcomes. The outer dashed lines indicate the triangular region within which 95% of studies are expected to be located in the absence of both biases and heterogeneity (fixed effect summary log odds ratio±1.96 × standard error of summary log odds ratio). The solid vertical line corresponds to no intervention effect (Sterne et al., 2011). The large samples are on the left, and the small samples are on the right.

Among these 22 studies, only four were eligible for the meta-analysis. These four studies were conducted in the West and measured students' perceptions of social relatedness (Berger and Hänze, 2009, *N* = 286; Costouros, 2020, *N* = 50; Hänze and Berger, 2007, *N* = 137; Roseth et al., 2019, *N* = 258). The overall ES appeared to be non-significant (*g* = 0.28, 95% CI [−0.15, 0.71], *p* = 0.19; see Table 5 and Figure 4) but to exhibit significant heterogeneity (*Q*(df = 3) = 19.98, *p* < 0.001, τ^2^ = 0.15, *I*^2^ = 88.33). More precisely, two studies concluded that the Jigsaw method had a non-significant effect on social relatedness (Roseth et al., 2019; Table 5, Berger and Hänze, 2009), whereas two studies (Hänze and Berger, 2007; Costouros, 2020) showed a positive effect.

**Table 5:** Estimated effect size and characteristics of students' relatedness.

**Study**	**Effect size**	**Confidence intervals**	**Sample**	**Study weight**	**Population**	**Discipline**	**Duration (hours)**
Hänze and Berger (2007)	0.87	[0.81, 0.93]	137	24.97	SS	Physics	9
Costouros (2020)	0.32	[−0.07, 0.20]	50	23.74	UNI	Management	48
Berger and Hänze (2009)	0.03	[−0.03, 0.09]	286	24.97	SS	Physics	9
Roseth et al. (2019)	−0.08	[−0.12, −0.04]	258	26.33	UNI	Anatomy	10
*K* = 3	0.28	[−0.15, 0.71]		100			

SS, Secondary school; UNI, University.

Figure 4 offers a visual representation of heterogeneity (i.e., publication bias).

#### 3.2.4. Student academic self-esteem

**Figure 5:**
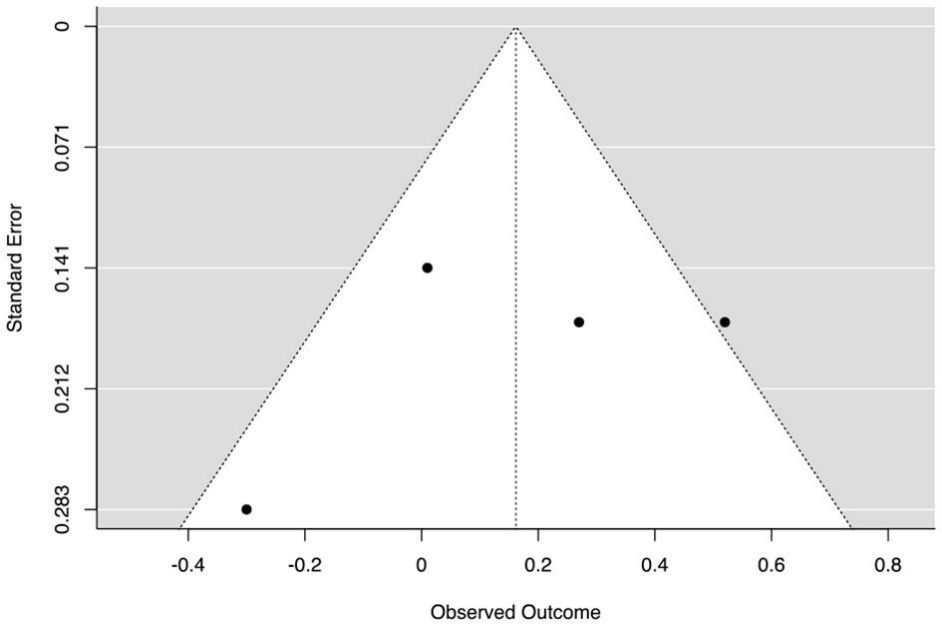
Funnel plot of the effect sizes of academic self-esteem outcomes. The outer dashed lines indicate the triangular region within which 95% of studies are expected to be located in the absence of both biases and heterogeneity (fixed effect summary log odds ratio ± 1.96 × standard error of summary log odds ratio). The solid vertical line corresponds to no intervention effect (Sterne et al., 2011). The large samples are on the left, and the small samples are on the right.

Among the six studies, four studies were eligible for the meta-analysis (Hänze and Berger, 2007; Berger and Hänze, 2009; Roseth et al., 2019; Costouros, 2020). The obtained ESs were small and nonsignificant (*g* = 0.25, 95% CI [−0.14, 0.46], *p* = 0.29; see Table 6 and Figure 5), and heterogeneity was observed (*Q*(df = 2) = 8.45 *p* = 0.04, τ^2^ = 0.06, *I*^2^ = 64.49).

**Table 6:** Estimated effect size and characteristics of students' academic self-esteem.

**Study**	**Effect size**	**Confidence intervals**	**Sample**	**Study weight**	**Population**	**Discipline**	**Duration (hours)**
Hänze and Berger (2007)	0.52	[0.46, 0.58]	137	26.54	SS	Physics	9
Berger and Hänze (2009)	0.27	[0.21, 0.33]	286	26.54	SS	Physics	9
Roseth et al. (2019)	0.01	[−0.03, 0.05]	258	29.88	UNI	Anatomy	10
Costouros (2020)	−0.30	[−0.85, 0.25]	50	17.04	UNI	Management	48
*K* = 3	0.16	[−0.14, 0.46]		100			

SS, Secondary school; UNI, University.

Figure 5 offers a visual representation of heterogeneity (i.e., publication bias).

### 3.3. Factors that could explain the heterogeneity of the results: moderating effects

The large dispersion and significant heterogeneity of each outcome can be explained by reference to the five factors previously identified as able to influence the Jigsaw effect.

#### 3.3.1. Sample size

For each outcome, the sample size appeared to be a factor that could explain the significant heterogeneity observed. Indeed, the ESs associated with students' achievement were larger (*Q*(df = 1) = 52.78, *p* < 0.001) in studies with sample sizes containing fewer than 102 participants (ES _*smallsamples*_ = 1.01, SE = 0.16, 95% CI [0.69; 1.33]) than in studies with sample sizes containing more than 102 participants (ES _*largesamples*_ = 0.33, SE = 0.23, 95% CI [−0.04; 0.70]). The mean sample size of studies reporting negative ESs was 224, whereas studies reporting large positive ESs had a mean sample size of 78 (Gömleksi'z, 2007; Göçer, 2010; Tarhan et al., 2013; Yapici, 2016). In addition, with regard to students' motivation, the two studies with the largest samples indicated small negative nonsignificant ESs (Berger and Hänze, 2009, *g* = −0.13; Roseth et al., 2019, *g* = −0.10), and two others reported positive significant ESs (Hänze and Berger, 2007, *g* = 0.35; Sanaie et al., 2019, *g* = 2.38) (see Table 4). The results pertaining to student motivation were similar when the four studies included in the meta-analysis were considered: the mean sample size of the studies with negative ESs was higher (*M* = 277, with the exception of Costouros (2020), which featured a sample size of 50 participants) than that of studies with positive ESs (*M* = 115.5). Moreover, the studies that found positive results regarding students' social relations had sample sizes ranging between 36 and 684 (*M* = 130). The studies that reported inconsistent results had the most important sample sizes (between 258 and 384 students, *M* = 297). Studies indicating negative effects had sample sizes ranging between 36 and 63. Finally, studies that reported positive results regarding students' self-esteem had smaller sample sizes (N_Moy_ = 103.5) than studies that reported non-significant results (N_Moy_ = 268). The studies on students' social self-esteem had consequent sample sizes (Blaney et al., 1977, p. 304; Lazarowitz et al., 1994, p. 120; Walker and Crogan, 1998, p. 103, *M* = 213).

#### 3.3.2. The discipline taught

For each outcome, the discipline taught did not appear to be a factor that could explain the significant heterogeneity observed. Indeed, the systematic review of students' achievement indicated positive effects of the Jigsaw method in many disciplines (sciences, English, literature, economics, etc.), although 78.38% of these studies focused on scientific disciplines. Moreover, no significant difference was observed among the ESs of studies investigating scientific disciplines [*Q*(df = 1) = 47.16, *p* = 0.15]. The same tendency can be observed in students' motivation and social relations, in which context 11 studies with positive results were conducted in different disciplines (i.e., sciences, literature), while ten studies found nonsignificant and negative results (i.e., sciences, physical education). Studies on social self-esteem were conducted in different academic disciplines in primary and secondary schools, and studies on academic self-esteem were conducted in the same discipline (i.e., the sciences).

However, the content taught appeared to be a factor that could explain the significant heterogeneity observed. Indeed, with regard to students' achievement outcomes, Berger and Hänze (2009) showed that the type of content taught leads to different Jigsaw effects. More precisely, Berger and Hänze (2015) showed that the content taught impacts the quality of teaching and therefore academic performance in the context of Jigsaw-based learning. The more demanding the content is, the higher the cognitive load faced by the student, and the more this factor hinders learning. The same result can be observed with regard to students' motivation outcomes (Berger and Hänze, 2015; Cochon Drouet et al., 2022).

#### 3.3.3. The students' diversity

For several outcomes, the students' diversity appeared to be a factor that could explain the significant heterogeneity observed. Indeed, in the study on physical education conducted by O'Leary et al. (2019), student diversity influenced the effect of the Jigsaw method on students' achievement. Indeed, those authors explained that the low ability of students negatively impacted their social and cognitive learning. Moreover, “the fact that higher-order social and cognitive learning was hampered for such students is likely to impact their psychomotor learning” with the Jigsaw method was noted (O'Leary et al., 2019, p. 724). Finally, some studies have shown that student diversity, particularly in students' ability, can impact their learning in the context of the Jigsaw method (O'Leary and Griggs, 2010; O'Leary et al., 2015, 2019) in a manner that disfavors students with low ability. The same tendency can be observed in the students' social relations and self-esteem outcomes. In fact, in three studies (O'Leary et al., 2015, 2019; Aydin and Biyikli, 2017), student diversity (e.g., ability level) was highlighted as a potential factor influencing the Jigsaw effect on students' social relations. As previously explained (see the Systematic Review section), students with low ability have more difficulty or generate more difficulties within their group. Moreover, according to Hänze and Berger (2007), students' diversity seems to be a factor that can affect the effects of the Jigsaw method on self-esteem in favor of students with low academic self-esteem as well as girls. However, no evidence was found to support the claim that student diversity (i.e., sex and status) influence the effect of the Jigsaw method on motivation (Hänze and Berger, 2007).

#### 3.3.4. The students' age

For all outcomes, the discipline taught did not appear to be a factor that could explain the significant heterogeneity observed. Indeed, the ESs associated with students' achievement were larger *Q*(df = 1) = 58.72, *p* = 0.62. Moreover, student age did not seem to explain the effect of heterogeneity on motivation, on students' social relations, or on students' self-esteem, and the relevant results showed positive and negative Jigsaw effects at different ages (primary and secondary schools vs. universities).

#### 3.3.5. The duration of implementation

For several outcomes, the duration of implementation did not appear to be a factor that could explain the significant heterogeneity observed. For studies on student achievement, the duration of implementation (< 9 h vs. >9 h, with a median implementation time of 9 h) did not lead to different ESs [*Q*(df = 1) = 53.59 *p* = 0.93], although the mean implementation time in the studies reporting positive ESs was 11 h, while the corresponding time in studies reporting negative ESs was 6 h. A similar conclusion could be drawn with regard to the impact of the implementation duration on students' motivation; this value was approximately 10 h in both studies reporting positive ESs and in studies reporting negative ESs [with the exception of Costouros (2020), which featured a duration of 48 h].

However, for studies on social relations and academic self-esteem, the duration of implementation appeared to be a factor that could explain the heterogeneity observed. In fact, concerning the duration of the implementation, studies that focused on students' social relations and reported a positive ES featured a mean Jigsaw practice time of 15 h, while studies that reported a negative ES featured a mean of 24 h of practice. Studies on students' self-esteem that reported positive results featured a mean Jigsaw practice of 10 h, and studies that reported non-significant results featured a mean of 24 h of practice.

## 4. Discussion

This paper proposed a mixed method review of studies that have investigated the effects of the Jigsaw method on students' outcomes. The objectives were (1) to determine the state of the art regarding these effects on students' educational outcomes precisely, (2) to improve our understanding of these effects, and (3) to highlight future lines of research and help teachers implement the Jigsaw method more effectively.

As a first output of this review, the 69 Jigsaw studies tested effects on three important types of educational outcomes: student learning, socialization, and self-esteem. Even if social relations were the historic goal of the Jigsaw method (Aronson et al., 1978), most studies focused on achievement.

### 4.1. Inconsistent and heterogeneous results

The primary result of our review pertains to the inconsistency reported regarding the effects of the Jigsaw method and the large variability observed among studies with regard to all retained student educational outcomes, with the exception of social self-esteem, for which only three studies found positive effects of the Jigsaw method (Blaney et al., 1977; Lazarowitz et al., 1994; Walker and Crogan, 1998). More precisely, concerning student achievement, the mean effect observed in our meta-analysis was positive and large but exhibited strongly significant heterogeneity. This heterogeneity in the meta-analysis occurred due to the variation in results among the included studies. Indeed, as explained in the introduction, studies conducted by reference to small samples tend to report unusually large effects and thus bias meta-analytic reviews (Kraft, 2020). Moreover, Funder and Ozer (2019) explained that situations in which small-sample studies report unusually large effect sizes may be a sign that the overall reliability is not trustworthy. Cheung and Slavin (2016) confirmed this claim. In their study, these authors showed that smaller-sample studies (*n* < 250) reported twice the ESs of larger-sample studies (*n* ≥ 250). Among the 17 studies reporting a large ES regarding achievement outcomes, the 16 studies from Turkey lack transparency (Stanczak, 2020), particular those that were conducted at the university. Some studies provided no information regarding the composition and selection of students included in the control and experimental groups. Nevertheless, Kyndt et al. (2013) explained that cooperative learning has stronger effects in “Eastern” societies, which are more collectivist, than in “Western” societies, which are more individualistic (Kitayama et al., 1997; Oyserman et al., 2002), and that this difference was statistically significant (δ = 0.38, 95% CI [0.25; 0.53]).

Concerning the motivation outcome, the results of the systematic review and the meta-analysis were ambivalent with regard to the possibilities of positive, non-significant or negative effects, indicating an overall non-significant ES and significant heterogeneity among studies with regard to both qualitative and quantitative analyses. As motivation is an antecedent of achievement, this result is consistent with the research on achievement that was previously discussed and provides additional evidence suggesting doubt regarding studies with small samples and very large positive ESs in terms of achievement. The students' social relations and academic self-esteem outcomes exhibit the same trend. From a qualitative perspective, the results regarding social relations are divided into two categories: positive results and non-significant or negative results. This result is particularly relevant since the Jigsaw method was initially created to enhance the social relations among students and to enable them to overcome prejudice between ethnic groups (Aronson et al., 1978). Moreover, the meta-analysis conducted by Johnson et al. (2007) showed that cooperative learning promotes social relations more effectively (*ES* = 0.68) than working alone (*ES* = 0.55). We develop the reasons for this unique characteristic of the Jigsaw method later in this discussion. Concerning social self-esteem, the results were predominantly positive. These results are very different from those concerning outcomes related to social relations. However, the studies conducted to investigate these two outcomes were not the same, and differences in the results concerning these theoretically associated variables can be explained by variability among studies.

More precisely, regarding this crucial point, even if a great deal of variability among studies is observed for nearly all types of outcomes, consistency is observed within studies that measure different educational outcomes simultaneously. For example, the ESs reported by Berger and Hänze (2009) with regard to achievement and motivation are consistent and in line with the theoretical link between those variables. Some studies have also presented consistent results regarding motivation and social relations (Hänze and Berger, 2007; Berger and Hänze, 2009; Roseth et al., 2019) according to the role of affiliative need completion in motivation (Deci and Ryan, 2000). This variability among studies and consistency within studies outlines the importance of contextual factors during the implementation of the Jigsaw method: the same Jigsaw structure can have positive or negative influences on educational outcomes depending on the context.

### 4.2. Factors influencing the effects of the Jigsaw method on educational outcomes

For different outcomes, the sample size seems to be a factor that influences the effects of the Jigsaw method. The discipline, the age of the students and the duration of implementation do not appear to be factors that influence the effects of the Jigsaw method on students. Finally, the content taught and the students' diversity are factors that influence the effects of the Jigsaw method.

#### 4.2.1. Sample size and the effect of the Jigsaw method

The results showed that ESs are influenced by the sample size pertaining to different outcomes: achievement, motivation and academic self-esteem. More precisely, the larger the ES is, the smaller the sample size pertaining to achievement outcomes in the meta-analysis. The positive large mean effect size appears to be driven by studies featuring underpowered small sample sizes and very high effect sizes. Studies on other educational outcomes are insufficient to support a similar analysis. However, we consistently observed that the mean sample size of studies that found that the Jigsaw method has a negative effect on motivation was higher than that of the studies that found a positive influence. For social relations and student academic self-esteem, studies reporting non-significant results had larger samples than those reporting positive significant effects. In fact, in the review, 60% of studies were underpowered, i.e., they featured a sample size < 102 (Maxwell, 2004; Stanczak, 2020). Moreover, these underpowered studies often reported a large or very large positive influence of the Jigsaw effect regardless of the outcomes. Finally, the observations made here regarding the Jigsaw method accurately represent the challenges posed by the replication crisis (Cheung and Slavin, 2016).

#### 4.2.2. Duration of implementation

In this review, the duration of the implementation did not appear to be a factor influencing the effects of the Jigsaw method on students. This factor was not relevant in the explanation of the heterogeneity observed in the results regarding student achievement. This result corroborated of the conclusions of the meta-analysis conducted by Stanczak (2020) regarding the effects of the Jigsaw method on student achievement, which showed that the efficacy of short-duration Jigsaw methods was not significantly different from that of longer-duration Jigsaw methods. This finding is consistent with our results concerning social relations and student academic self-esteem. This observation contradicts Aronson and Patnoe's (2011) assertion that cooperation in a Jigsaw context takes time to develop before gains in learning are observed (Stanczak, 2020). An implementation that features a duration longer than 9 h may lead to fatigue, boredom and a decrease in motivation, thus reducing learning gains. Indeed, students appreciate the changes. Similarly, Lentillon-Kaestner and Patelli (2017) showed that students perceived higher pleasure when the grouping forms changed regularly during a physical education sequence. The results reported by Cochon Drouet et al. (2022) are in line with Aronson's claim. Indeed, this study showed that the Jigsaw effects on motivation and moderate to vigorous physical activity increased over time in the context of physical education (between lessons 3 to 6) regardless of whether those effects were positive or negative. The impact of the duration of implementation remains unclear and requires further investigation.

#### 4.2.3. The type of content taught and student diversity

In this review, discipline did not appear to be a factor that could influence the effects of the Jigsaw method on students. Among the studies on achievement included in this meta-analysis, 78.38% focused on scientific disciplines, but no significant difference was observed between ESs between studies conducted in the context of a scientific discipline and those conducted in the context of other disciplines. The meta-analysis conducted by Stanczak (2020) on the effects of the Jigsaw method on achievement showed similar results. However, our review revealed that the effects of the Jigsaw method could vary with the content taught (Hänze and Berger, 2007; Berger and Hänze, 2009; Theobald et al., 2017; Cochon Drouet et al., 2022). For example, the results reported by Cochon Drouet et al. (2022) with regard to physical education showed that the effect of the Jigsaw method on student motivation can be positive or negative depending on the type of sport taught during the implementation process. By focusing on this question, Berger and Hänze (2015) highlighted the difficulty of teaching pupils some subtopics in physics using the Jigsaw method. Complex topics result in a high intrinsic cognitive load and hinder the quality of expert instruction and thus partner learning. The difficulty of teaching impacts student learning and achievement. This factor also makes it more difficult to establish positive interdependence and can undermine social relations. In summary, even if the nature of the discipline does not influence the effect of the Jigsaw method significantly, the type of content taught does. Further studies should take this point into account. However, such a conclusion highlights the important effect of context and the importance of the content taught using the Jigsaw method, which seems to influence the outcomes of implementation and could explain the observed variability in the Jigsaw effect across studies.

Consistent with these observations regarding the complexity of the content that students must learn and then teach, student diversity (e.g., level of ability, self-concept and sex) appeared to influence the Jigsaw effect on achievement, social relations, and academic self-esteem (Hänze and Berger, 2007; O'Leary and Griggs, 2010; Berger and Hänze, 2015; O'Leary et al., 2015, 2019). In fact, very often, the lower students' levels are, the more difficulties they face, especially in social relations (O'Leary et al., 2015, 2019). Thus, the fit between the complexity of the content to be taught and student ability appears to be important for avoiding problems when using the Jigsaw method and preventing negative consequences on students' achievement and relationships (O'Leary et al., 2015, 2019). When the complexity of the content taught fits with students' resources, the Jigsaw method can be more beneficial for students with low resources. For example, the study conducted by Vives (2021) showed that the Jigsaw method had benefits in terms of academic performance only for students with low self-esteem and low working memory capacity. Teachers must be specifically trained in these different elements to include all students when implementing the Jigsaw method.

### 4.3. Ways to improve the implementation of the Jigsaw method

The results of this review highlighted factors and conditions that enable the Jigsaw method to “work” to achieve positive effects in terms of student achievement, motivation, social relations and self-esteem. Roseth et al. (2019) emphasized the fact that the Jigsaw method is a structure that generates various types of relationships among individuals. The phases of the expert group and coteaching appear to be particularly sensible with regard to the implementation of the Jigsaw method and could be sources of heterogeneity among studies (Roseth et al., 2019).

#### 4.3.1. The expert groups

As explained by Roseth et al. (2019), the expert phase can lead to individualistic behaviors and therefore a loss of cohesion that is unfavorable to group dynamics. Problems of status within a class can also emerge, as it is difficult for a low-ability student to master the proposed content and attain credibility as an expert, leading to problems in the expert phase that could explain the variability in the effect of the Jigsaw method between studies. To overcome this issue, Roseth et al. (2019) suggested breaking down the stages of the Jigsaw method to identify the processes when students are involved in expert or Jigsaw groups. Ensuring that students exhibit interdependence with regard to resources should be a prerequisite. The time spent in the expert group could be lengthened by a phase of preparation that can allow students to reflect on and practice how they might teach the material prior to teaching their peers in their home group; this reflection could be based on some explicit prompts such as “defend your answer”, “put yourself in your friend's shoes” or “probe your groupmates for justification” (Theobald et al., 2017). Teachers can also provide specific assistance and verify that low-achieving students are mastering the skills that they will need to teach their classmates in the coteaching phase, a point of which teachers should be made aware during Jigsaw training.

#### 4.3.2. The coteaching phase

The coteaching step is also a phase that can lead to group problems because students must understand the content in a limited amount of time and find a way to teach it in a manner that other students can understand. The Jigsaw method is very demanding for students. Some studies in the present review highlighted the problems of conflicts associated with the coteaching step of the Jigsaw method (O'Leary and Griggs, 2010; O'Leary et al., 2015; Aydin and Biyikli, 2017). While group work can be extremely fruitful, it can also be ineffective (Slavin, 2011).

Problems that emerge during the coteaching step can lead to a negative interdependence between students and even a competence threat (Buchs et al., 2018) if the expert of the group does not play his or her role. Indeed, the informational dependence that emerges in this phase of the Jigsaw method may be problematic for learning if the information is of poor quality (Buchs et al., 2004, 2010, 2018). This kind of competence threat is likely to reduce learning (Buchs et al., 2010; Buchs and Butera, 2015) and motivation as well as to result in less-constructive interactions with others (Buchs et al., 2004, 2018; Buchs and Butera, 2009).

To complete this phase successfully, the main content chosen for group work should be divided into subcategories to ensure that all group members have equal responsibilities (Karacop and Doymus, 2013; O'Leary et al., 2015). Indeed, as is the aim of the logic of the Jigsaw method, the resources must be interdependent and complementary. “The positive relationship between a partner's competence and students' learning is found only when students work on complementary information” (Buchs et al., 2018, p. 2).

This coteaching phase must therefore be made easier or spread out over time and supervised by the teachers in greater depth. This task can include training students to teach their peers over the course of several lessons before implementing the Jigsaw method. “Ovens et al. (2012) recommend that pupils are given additional time to consider how they might teach material to their peers” (O'Leary et al., 2015, p. 189). Finally, this coteaching phase can be effective only if all pupils have satisfactorily succeeded in the expert step of the Jigsaw method.

#### 4.3.3. Time spent training teachers

The variability obtained highlights the importance of context; the best way to control all these contextual effects is therefore for teachers to receive sufficient training to anticipate and manage the problems associated with the Jigsaw method (Drouet et al., 2020). This development entails the appropriation of these principles and their application to the teaching content. The studies included in this review remain evasive on this point, which is crucial. Bratt (2008) offered 2 days of training. Several studies have followed these recommendations (Roseth et al., 2019; Cochon Drouet et al., 2022, 2023), and future implementations would benefit from following them. The time spent training teachers could alter the effects of the Jigsaw method. Cochon Drouet et al. (2023) showed that Jigsaw training for teachers helps facilitate professional development and encourages changes in practice that are beneficial to all students, especially those facing difficulties.

## 5. Limitations and directions for future research

A first limitation of this review is that some studies featuring many dependent variables appear in several variable analyses (e.g., achievement, motivation, social relations) and are weighed heavily in our results, especially in our meta-analyses. Moreover, meta-analysis could not always be performed due to the lack of statistical data, the lack of studies or differences in the constructs measured regarding the same theme. Finally, some meta-analyses were conducted by reference to only a few studies. Even if meta-analyses are particularly transparent and more likely to be valid than other synthesizing techniques (Valentine et al., 2010), ES estimates drawn from small samples are more sensitive to sampling error, which affects their precision and increases the likelihood of reporting extreme estimates (Kühberger et al., 2014). Therefore, these meta-analyses must be interpreted cautiously, although they complement our systematic review.

Second, in our systematic review, we followed the PRIMA guidelines (Page et al., 2021). However, we could have followed the guidelines proposed by Risko et al. (2008) and Torgerson et al. (2005), as Scott et al. (2018) to verify the methodological quality of their studies in a systematic review in light of the seven indicators included in the Methodological Quality Questionnaire.

Third, the findings of the review should be interpreted in the context of potential publication bias (Sutton et al., 2000; Thornton and Lee, 2000). Publication bias refers to the tendency of researchers and journal editors not to publish studies that fail to find significant effects. This paper is based only on published studies, and this limitation must be considered when interpreting our results. Therefore, the inconsistency regarding the effects of the Jigsaw method on student learning, social relations and self-esteem outcomes observed in this context should be even more frequent than explained in this meta-analysis. Moreover, the mean ES measured here would certainly be even smaller if studies that were not published were considered. Future research should focus on an appropriate sample size (> 176 participants for a 95% chance of detecting the mean effect) and should further test potential moderators of the effect of the Jigsaw method. According to our observations in this review, prospects for research with large samples are increasing, particularly with regard to clearly identifying the moderating effects of student diversity and duration of implementation. It would also be interesting to analyze the problematic phases of the Jigsaw method in further detail and to test this method in the context of the adaptations of the elements highlighted previously.

## 6. Conclusion

The main result of our systematic review and meta-analyses pertains to the inconsistency of the effects of the Jigsaw method on students' educational outcomes, despite the fact that 69 studies have investigated this topic. This inconsistency corresponds to a strong variability and even ambivalence among these studies, revealing that the Jigsaw method is very dependent on contextual influences. The same pedagogical structure, which is quite rigid and seemingly easy to implement, can have diverse effects depending on contextual elements. Rather than asking for and simply reproducing an invariant structure independent of the context, teachers must think carefully about possible interactions between their teaching contexts and this structure; they should also complete sufficient relevant training. Moreover, our qualitative review identified some contextual factors that modify the Jigsaw effect (i.e., the content taught, student diversity). These results can help teachers and teacher-trainers focus on the decisive element when improving the effects of the Jigsaw method.

From a methodological perspective, these results highlight the complementarity of meta-analytic and systematic qualitative review approaches with regard to investigating the Jigsaw effect. Significant meta-analytic results help confirm global results. A qualitative review helps improve our understanding of such global results. For example, if our meta-analyses clearly indicated the significant variability of the Jigsaw effect regardless of the outcomes considered (i.e., achievement, motivation, social relations and self-esteem), the quantitative test of a priori factors that could explain this variability remained inconclusive, with the exception of those pertaining to sample size. Our qualitative examination of the literature helped us identify the factors that can explain this variability more accurately. This approach helped us progress from two independent questions about the impacts of diversity and the discipline taught on the Jigsaw effect to one more heuristic focus on the fit between the complexity of the content taught and the diversity of students' resources.

Finally, in light of the significant heterogeneity of results, the very large effect size observed in some studies and the significant influence of sample sizes on Jigsaw effects, the literature on the Jigsaw method appropriately represents the challenges posed by the replication crisis (Cheung and Slavin, 2016). This result highlights the need for higher-quality studies, preregistrations and careful research questions. Beyond improving “Jigsaw implementation” *per se*, such a perspective is necessary to overcome the difficulties posed by the replication crisis. Researchers must also focus on and investigate the processes that occur during the implementation of a pedagogical structure in other contexts, especially those pertaining to the use of the Jigsaw method during the expert group and coteaching phases, which appeared in our review as potentially decisive phases with regard to the Jigsaw method's effect. Cooperative methods can promote inclusive education only if access to this cooperative learning is guaranteed to all; this goal can be achieved through thoughtful teacher training (Drouet et al., 2020).

## Data availability statement

The original contributions presented in the study are included in the article/Supplementary material, further inquiries can be directed to the corresponding author.

## Author contributions

OD organized the database, performed the statistical analysis, wrote the first draft, and sections of the manuscript. All authors contributed to manuscript revision, read, and approved the submitted version.
