# Supplementary material for: Effects of the Jigsaw method on student educational outcomes: systematic review and meta-analyses
Source: Front Psychol. 2023 Aug 3;14:1216437. doi: 10.3389/fpsyg.2023.1216437 (PMC10436097; doi:10.3389/fpsyg.2023.1216437)
Supplement: Supplementary file 1 [file Table_1.docx]

**Appendix**

*Complete Description of the Selected Studies*

| Study | Journal | SJR | *N total* | Geographic location | Jigsaw method | Discipline | Population | Duration | Age [y] | Percent of boys | Outcome measures | Results | ES *(var)* |
| --- | --- | --- | --- | --- | --- | --- | --- | --- | --- | --- | --- | --- | --- |
| *Abed et al., 2020 | International Electronic Journal of Mathematics Education | Q3 | 80 | Iraq | Jigsaw I | Math | Secondary school | 8 hours (8 weeks) | NM | 0 | General Achievement test | The Jigsaw increased achievement | A: 1.49 (*.06*) |
| *Akkus & Doymus, 2022 | Journal of Turkish Science Education | Q2 | 68 | Turkey | Subject Jigsaw method | Sciences | Primary school | NM | NM | 52.7 | Achievement test: quiz  Interviews toward instructional teaching method | Jigsaw increased achievement. Students had developed positive attitude toward the Jigsaw method. |  |
| *Arslan, 2016 | Educational Research and Reviews | Q4 | 56 | Turkey | Jigsaw I | Turkish | Secondary school | 12 hours (3 weeks: 3 class hours/week) | NM | 51.8 | Achievement test : academic success test  Attitude scale of Turkish courses | No significant difference on achievement. | A: .38 (.*07*) |
| *Artut & Tarim, 2007 | Asia Pacific Journal of Education | Q1 | 71 | Turkey | Jigsaw II | Math | University (future teachers) | 36 hours (9 weeks) | NM | 44.4 | Achievement test  Perceptions: Opinion’s survey  Diaries observation | The Jigsaw II increased achievement | A: 1.09 *(.06)* |
| *Aydin & Biyikli, 2017 | Universal Journal of Educational Research | Q4 | 63 | Turkey | Jigsaw I | Physics | University | 12 hours (6 weeks: two class hours/week) | NM | NM | Achievement test: laboratory skill evaluation scale  Perceptions: Jigsaw Opinion Scale | The Jigsaw increased achievement. | A: 1.31 (.07) |
| Study | Journal | SJR | *N total* | Geographic location | Jigsaw method | Discipline | Population | Duration | Age [y] | Percent of boys | Outcome measures | Results | ES *(var)* |
| Azmin, 2015 | International Education Studies | Q3 | 16 | Turkey | Jigsaw I | Psychology | University (college) | NM | 18.13 | 43.75 | No control group.  Achievement test (knowledge and evaluation skills): pre-post test  Perceptions: Opinion’s survey in the Brunei context | Jigsaw increased achievement.  Students had developed positive attitude toward the Jigsaw method. | NA |
| Baken et al., 2022 | Journal of Biological Education | Q2 | 1970 | United-States | Jigsaw I | Biology | University | One semester | NM | NM | Achievement test: quiz | Jigsaw increased achievement. | NA |
| *Berger & Hänze, 2009 | International Journal of Science Education | Q1 | 286 | Germany | Jigsaw I | Physics | Secondary school | 9 hours | NM | NM | Learning experience questionnaire competence, autonomy, relatedness, intrinsic motivation)  Achievement test | No significant difference on achievement, motivation and relatedness. | A: -0.07 *(.02)*  M: -0.13 *(0.03)*  R*: 0*.03 *(.03)*  ASE: 0.27 *(.03)* |
| Berger & Hänze, 2015 | International Journal of Science Education | Q1 | 129  (No control group) | Germany | Jigsaw I | Physics | Secondary school | 6 hours | NM | 67 | Intrinsic motivation  Achievement performance  Perception of teaching quality | Academic performance of novice students increases with the quality of expert students’ instruction. Higher instructional quality of more difficult subtopics did not lead to better academic performance of novice students. |  |
| Study | Journal | SJR | *N total* | Geographic location | Jigsaw method | Discipline | Population | Duration | Age [y] | Percent of boys | Outcome measures | Results | ES *(var)* |
| Blajvaz et al., 2022 | Journal of Baltic Science Education | Q2 | 92 | Serbia | Jigsaw I | Physics | Secondary school | One semester | 13 | 58.69 | Achievement test  Motivation  Metacognition awareness | Jigsaw increased achievement, motivation and metacognitive awareness. | NA |
| Blaney et al., 1977 | Journal of Educational Psychology | Q1 | 304 | United States | Jigsaw I | NM | Primary school | 18 hours (6 weeks) | NM | NM | Attitude questionnaire (peer liking, linking for school, self-esteem)  Sociometric instrument | Students in Jigsaw manifested higher self-esteem than in controls and liked groupmates more than other classmates. Black and Anglo of Jigsaw increased their liking for school more than blacks and Anglos in control. | NA |
| Bratt, 2008 | Journal of Community & Applied Social Psychology | Q1 | Study 1: 68  Study 2: 164 | Norway | 1 | NM | Secondary school | Study 1: 7 weeks (2h/week)  Study 2: 8 weeks (1 or 2h/week) | Study 1: 11  Study 2: 14 | NM | Study I: attitudes toward classmates and school; empathy.  Study II: attitude toward classmate; identity; empathy | No effect on intergroup relationships; Decrease of empathy levels | NA |
| Study | Journal | SJR | *N total* | Geographic location | Jigsaw method | Discipline | Population | Duration | Age [y] | Percent of boys | Outcome measures | Results | ES *(var)* |
| Bridgeman, 1981 | Child Development | Q1 | 120 | United States | Jigsaw I | NM | Primary school | 8 hours (8 weeks) | 10.2 | 44.16 | Role taking measure  Moral reasoning | Role taking was found to be enhanced by cooperative interdependence. No significant result on moral reasoning level. | NA |
| Chang & Benson, 2022 | Innovations in Education and Teaching International | Q1 | 42 | 17 countries | Jigsaw I | Management | University | One semester | 25 | NM | No control group. Online questionnaire comprising five sections: personal information, cultural diversity, perceived usefulness of cloud applications, jigsaw satisfaction, and collaboration. | Jigsaw influenced positively individual learning in the group. | NA |
| Cochon Drouet et al., 2022 | Journal of Teaching Physical Education | Q1 | 136 | Switzerland | Jigsaw 1 | Physical Education | Secondary school | 24 hours (16 weeks) | 14.11 | 51 | Moderate to vigorous physical activity (MVPA)  Motivation: Situational Interest (SI) | Jigsaw progressively enhanced MVPA and the novelty dimension of SI in gymnastics, whereas it undermined MVPA and the challenge dimension of SI in rackets. |  |
| Study | Journal | SJR | *N total* | Geographic location | Jigsaw method | Discipline | Population | Duration | Age [y] | Percent of boys | Outcome measures | Results | ES *(var)* |
| *Costouros, 2020 | Teaching and Learning Inquiry | Q3 | 50 | Canada | Jigsaw I | Business Management | University | 48 hours (12 weeks) | 21 | 54.00 | Achievement test: quiz in post-test  Survey on student perceptions on their experiences of autonomy, competence, social relatedness, intrinsic motivation, and deeper-level processing. | No significant difference on the results of students’ achievement, autonomy, competence and intrinsic motivation. Jigsaw increased social relatedness and deeper-level processing for international students only. | A: 0.18 *(.04)*  M: -0.04 *(.04)*  R: 0.32 *(.04)*  SE: -0.30 (.08) |
| Crone & Portillo, 2013 | Teaching of Psychology | Q2 | 35 | United States | Jigsaw I | Psychology | University | 12 hours (1 semester) | 26.39 | 15.7 | Attitudinal measures | Jigsaw’ students report an increased ability to teach psychological concepts to other students. | NA |
| Desforges et al., 1991 | Journal of Personality and Social Psychology | Q4 | 95 | United States | Jigsaw I | Psychology | University | 2 hours | NM | 27.37 | Achievement test : assessment of learning  Attitude surveys  Impressions | Students had developed positive attitude and more empathy about a typical mental patient. |  |
| Study | Journal | SJR | *N total* | Geographic location | Jigsaw method | Discipline | Population | Duration | Age [y] | Percent of boys | Outcome measures | Results | ES *(var)* |
| *Doymus, 2007 | Journal of Chemical Education | Q2 | 108 | Turkey | Subject Jigsaw | Chemistry | University | 12 hours (3 weeks) | NM | NM | Chemical equilibrium achievement test | The Jigsaw increased achievement. | A: 0.96 *(.05)* |
| *Doymus, 2008a | Research in Science Education | Q1 | 36 | Turkey | Subject Jigsaw | Chemistry | University | 9 hours (3 weeks) | NM | NM | Chemical equilibrium achievement test | The Jigsaw increased achievement. | A: 1.04 (.08) |
| *Doymus, 2008b | Research in Science and Technological Education | Q2 | 68 | Turkey | Subject Jigsaw | Chemistry | University | 10 hours (5 weeks) | NM | NM | Chemical equilibrium achievement test | The Jigsaw increased achievement. | A: 1.75 (.20) |
| *Doymus et al., 2010 | Educational Technology Research and Development | Q1 | 122 | Turkey | Subject Jigsaw | Chemistry | University | 5 hours (5 weeks) | NM | NM | Achievement test: test of scientific reasoning and particulate nature of matter evaluation test | The Jigsaw increased achievement. | A: 1.47 (.08) |
| *Er, 2017 | Universal Journal of Educational Research | Q4 | 46 | Turkey | Jigsaw I | Social | Secondary school | 12 hours (4 weeks) | NM | 58.7 | Achievement test  Interviews Form | The Jigsaw increased achievement  Students had positive statements about Jigsaw. | A: 1.14 (.15) |
| Gambari & Yusuf, 2017 | Journal of Peer Learning | Q4 | 167 | Nigeria | Jigsaw II | Physics | Secondary school | Six weeks | NM | NM | Physics Achievement test  Physics Attitude scale | Jigsaw increased achievement. | NA |
| *Garcia, 2021 | Education and Information Technologies | Q1 | 80 | Philippines | Jigsaw I | Computer programming | University | 14 weeks | 19.08 | 93.75 | Achievement test: quiz  Attitude scale of computer programming learning and computer programming self-efficacy scale | Jigsaw increased achievement, attitude toward learning and self-efficacy of students. | A: 0.45 *(.05)* |
| Study | Journal | SJR | *N total* | Geographic location | Jigsaw method | Discipline | Population | Duration | Age [y] | Percent of boys | Outcome measures | Results | ES *(var)* |
| *Ghaith & El-Malak, 2004 | Educational Research and Evaluation | Q2 | 48 | Liban | Jigsaw II | Reading | Secondary school | 15 hours (five weeks) | 18 | 60.4 | Achievement test | The Jigsaw  increased achievement. | A: 0.27 (.09) |
| *Göçer, 2010 | Educational Research and Reviews | Q4 | 60 | Turkey | Jigsaw I | Literature | Secondary school | 3 hours (3 weeks) | NM | NM | Achievement test: Genre Questions List  Interviews | The Jigsaw  increased achievement. Students had a positive opinion about Jigsaw. | A: 4.29 (.21) |
| *Gömleksi˙ z, 2007 | European Journal of Engineering Education | Q1 | 66 | Turkey | Jigsaw II | English | University | 28 hours (2 class hour/week during 14 weeks) | 21.47 | NM | Achievement test: Questions  Attitude questionnaire | The Jigsaw  increased achievement and attitudes towards learning English. | A: 2.38 *(.15)* |
| Halimah & Sukmayadi, 2019 | International Journal of Instruction | Q2 | 60 | Indonesia | Jigsaw I | Pedagogy for preservice teachers | University | 23 hours (one semester) | NM | NM | Observation, field note and interviews to explore the role of Jigsaw on pedagogical content knowledge and verbal communication skills | The results show that Jigsaw improves both the pedagogical knowledge and verbal communication skills of preservice teachers. | NA |
| *Hänze & Berger, 2007 | Learning and Instruction | Q4 | 137 | Germany | Jigsaw I | Physics | Secondary school | 9 hours | NM | NM | Learning experience questionnaire (3 types: competence, autonomy, social belonging, intrinsic motivation)  Achievement test | No significant difference on the results of students’ achievement.  Positive differences in students’ experience of competence, social relatedness and autonomy, and in intrinsic motivation in favor of Jigsaw group. | A: -0.24 *(.03)*  M: 0.35 *(.03)*  R: 0.87 *(.03)*  ASE: 0.52 *(.03)* |
| Study | Journal | SJR | *N total* | Geographic location | Jigsaw method | Discipline | Population | Duration | Age [y] | Percent of boys | Outcome measures | Results | ES *(var)* |
| *Hornby, 2009 | Journal of Education for Teaching | Q1 | 44 | New Zealand | Jigsaw I | Education | University | 2 hours | 22 | 6.8 | Achievement test: multi-choice test on cooperative learning  Attitude: questionnaire | The Jigsaw increased achievement. | A: .76 *(.09)* |
| *Karacop & Doymus, 2013 | Journal of Science Education and Technology | Q1 | 115 | Turkey | Subject Jigsaw | Chemistry | University | 5 hours (5 weeks) | NM | NM | Achievement test: test of scientific reasoning, purdue spatial visualization of rotation test, the chemical bonding academic achievement test, particulate nature of matter test in chemical bonding | The Jigsaw increased achievement. | A: 1.09 *(.09)* |
| *Kilic, 2008 | World Applied Sciences Journal | Q4 | 80 | Turkey | Jigsaw I | Pedagogy | University | NM | NM | NM | Achievement test: Program Development Success Test | Jigsaw increased achievement. | A: 1.13 *(.05)* |
| *Koç et al., 2010 | Journal of Turkish Science Education | Q2 | 106 | Turkey | Subject jigsaw | Chemistry | University | 16 hours (4h/week during 4 weeks) | 19.44 | NM | Chemic Kinetics achievement test and Graphic skills test | The Jigsaw increased achievement. | A: 1.90 *(.07)* |
| Study | Journal | SJR | *N total* | Geographic location | Jigsaw method | Discipline | Population | Duration | Age [y] | Percent of boys | Outcome measures | Results | ES *(var)* |
| *Koç et al., 2016 | Educational Research and Reviews | Q2 | 71 | Turkey | Jigsaw II | Science | Secondary school | 20 hours (4 class hours/ week, 5 weeks | NM | NM | Academic achievement est  Attitude: Science lesson attitude scale | No significant difference on achievement. | A: 0.53 *(.09)* |
| Lazarowitz et al., 1994 | Journal of Research in Science Teaching | Q1 | 120 | Israel | Jigsaw I | Sciences | Secondary school | 5 hours (5 weeks) | NM | NM | Achievement test  Attitude  Creativity  Self-esteem  Relationships | The Jigsaw increased achievement.  Jigsaw‐group mastery learning students scored significantly higher on self‐esteem, number of friends, and involvement in the classroom. No difference was found in cohesiveness, cooperation, competition, creativity and attitudes toward the subject learned. | NA |
| Legrain et al., 2019 | Physical Education and Sport Pedagogy | Q1 | 69 | France | Jigsaw I | Physical education | University | 6 hours (3 weeks) | 21 | 66.7 | Teacher’s motor skills  Knowledge for practice Pedagogical knowledge  Perceived self-efficacy | No difference was found in self-efficacy. The Jigsaw increased teaching skills test. | NA |
| Study | Journal | SJR | *N total* | Geographic location | Jigsaw method | Discipline | Population | Duration | Age [y] | Percent of boys | Outcome measures | Results | ES *(var)* |
| Lucker et al., 1976 | American Educational Research Journal | Q1 | 305 | United States | Jigsaw I | NM | Primary school | 1h30 (2 weeks: 45min daily) | NM | NM | Achievement test | Results showed that Anglos perform equally well in both conditions. Minorities performed significantly better in Jigsaw than control classes. | NA |
| Maden, 2011 | Educational Sciences: Theory and Practice | Q3 | 70 | Turkey | Jigsaw I | Turkish | University | 24 hours (6 weeks: 4 class hours/week) | NM | NM | Kolb learning style inventory  Achievement test: success test for written expression  Students’ view form | No significant difference on achievement. Students had positive Jigsaw views. | NA |
| Maison et al., 2021 | International Journal of Instruction | Q2 | 55 | Indonesia | Jigsaw I | Physics | Secondary school | NM | NM | NM | Students’ attitude toward physics: survey and interviews | Students had positive perceptions toward physics after the Jigsaw implementation. | NA |
| Mari & Gumel, 2015 | International Journal of Information and Education Technology | Q3 | 200 | Nigeria | Jigsaw I | Chemistry | Secondary school | NM | NM | NM | Achievement test: quiz  General self-efficacy scale. | Jigsaw increased achievement for formal reasoners. No significant difference on self-efficacy. | NA |
| Study | Journal | SJR | *N total* | Geographic location | Jigsaw method | Discipline | Population | Duration | Age [y] | Percent of boys | Outcome measures | Results | ES *(var)* |
| *Moreno, 2009 | Learning and Instruction | Q1 | 87 | United States | Jigsaw (assisted by computer) | Biology | University | 1 hour | 24.29 | 63.3 | Achievement test  Learning perceptions | Results showed no difference in achievement. Students had positive Jigsaw’ perceptions. | A: -0.65 *(.07)* |
| Moskowitz et al., 1983 | American Educational Research Journal | Q1 | 261 | United States | Jigsaw I | NM | Primary school | 80 hours (1 year: 2 hours/ week) | NM | 35.63 | Achievement test  Affective teaching climate  Attitudes toward school  Academic and social self-esteem Attitudes toward peers | Few affective gains were found although participants in Jigsaw rated their classes as less competitive | NA |
| Moskowitz et al., 1985 | Contemporary Educational Psychology | Q1 | 384 | United States | Jigsaw I | NM | Primary school | 80 hours (1 year: 2 hours/ week) | NM | NM | Attitudes toward self, peers, and school  Achievement test and attendance records | Jigsaw failed to have a positive effect on the outcome variables. | NA |
| *Namaziandost et al., 2020 | Cogent Arts & Humanities | Q3 | 50 | Iran | Jigsaw I | English | Secondary school | 20 hours: 20 sessions | 17 | 100 | Achievement test | The Jigsaw increased achievement. | A: 1.07 *(.09*) |
| Oakes et al., 2019 | Anatomical Sciences Education | Q1 | 145 | Australia | Jigsaw I | Anatomy | University | 2 hours | 20.3 | 23.4 | Students’ perception of the Jigsaw method  Achievement test | Results showed no difference in achievement.  Participants perceived the jigsaw as highly for both educational value and enjoyment and felt the teaching approach would improve their course performance. | NA |
| Study | Journal | SJR | *N total* | Geographic location | Jigsaw method | Discipline | Population | Duration | Age [y] | Percent of boys | Outcome measures | Results | ES *(var)* |
| O’Leary & Griggs, 2010 | Journal of Further and Higher Education | Q2 | 61 | England | Jigsaw I | Physical education | University | 4 hours (4 weeks) | NM | NM | Focus-group interviews and field notes during 4 weeks | Efficacy of the method on the cognitive and affective domain. The Jigsaw was not seen to be very effective for psychomotor learning. | NA |
| O’Leary et al., 2015 | European Physical Education Review | Q1 | 62 | England | Jigsaw I | Physical education | Secondary school | 8 hours | 15 | 0 | Observations  Reflective journals | Results indicated some problems with the students as peers teaching or group management. | NA |
| O’Leary et al., 2019 | European Physical Education Review | Q1 | 36 | England | Jigsaw I | Physical education | University | 48 hours (12 weeks: four hours/week) | NM | 53 | Focus group interviews Reflective journals | Students felt that heterogeneous and friendship groupings have the potential to encourage high-order social and cognitive learning. | NA |
| *Roseth, Lee, & Saltarelli, 2019 | Journal of Educational Psychology | Q1 | 258 | United States | Jigsaw I | Anatomy | University | 10 hours (8 weeks x75min) | 21 | 32 | Achievement test; Social interdependence scale; Sociocognitive conflict regulation; Perceived competence; Relatedness; Interest | The only significant differences between group were for relational regulation and academic achievement. | A: 0.26 *(.01)*  R: -0.08 *(.02)*  ASE: 0.01 *(.02)* |
| Study | Journal | SJR | *N total* | Geographic location | Jigsaw method | Discipline | Population | Duration | Age [y] | Percent of boys | Outcome measures | Results | ES *(var)* |
| *Sahin, 2010 | Educational Research and Reviews | Q4 | 80 | Turkey | Jigsaw II | Turkish | University | 24 hours (6 weeks: four hours/week) | NM | NM | Achievement Test  Attitude  Students to written expression course | The Jigsaw increased achievement, retention and attitude. | A: 0.97 *(.05)* |
| *Sahin, 2011 | Asia Pacific Education Review | Q2 | 71 | Turkey | Jigsaw III | Writing | Primary school | 24 hours (6 weeks: 4h/week) | NM | 45.0 | Achievement test in Turkish course  Opinion questionnaire | The Jigsaw increased achievement. | A: 0.86 (.06) |
| *Sanaie et al., 2019 | Nurse Education Today | Q1 | 94 | Iran | Jigsaw I | Nursery | University | 34 hours (1 semester 17x2 hours) | NM | NM | Self-regulated learning questionnaire Motivation strategies | The Jigsaw increased self-regulated learning and academic motivation. | M: 2.38 (.07) |
| Santos Rego & Del Mar Lorenzo Moledo, 2005 | Intercultural Education | Q3 | 250 | Spain | Jigsaw I | NM | Secondary school | 3 hours (3 weeks) | NM | NM | Attitude | No significant difference for attitude. |  |
| *Sagsoz et al., 2015 | European Journal of Dental Education | Q2 | 50 | Turkey | Jigsaw I | Dentistry | University | 3 weeks | NM | NM | Achievement test | No significant difference on achievement. | A: 0.34 *(.07)* |
| Study | Journal | SJR | *N total* | Geographic location | Jigsaw method | Discipline | Population | Duration | Age [y] | Percent of boys | Outcome measures | Results | ES *(var)* |
| *Shaaban, 2006 | Reading Psychology | Q2 | 45 | Liban | Jigsaw II | Reading | Secondary school | 8 hours (8 weeks: 60min/week) | 11.45 | 59.1 | Achievement test: Gates-Mcginitie reading test  Motivation to read profile | The Jigsaw increased motivation. No significant difference for achievement. | A: 0.23 *(.13)*  M: 1.58 *(.11)* |
| *Souvignier & Kronenberger, 2007 | British Journal of Educational Psychology | Q1 | 208 | Germany | Jigsaw I | Sciences (math-astronomy) | Primary school | 15 hours (2x6 lessons of 45minutes) | 8.11 | 48.1 | Achievement test | There were no differences between group in maths. | A: -0.48 *(.02)* |
| Stanczak et al., 2022 | Journal of Educational Psychology | Q1 | Study A: 252  Study B: 313  Study C: 110  Study D: 74  Study E: 101 | France | Jigsaw I | Sciences | Secondary school | A and b: 2h  C: 16h  D and e: 18h | 11.6 | A: 41.66  B: 46  C: 52.73  D: 48.65  E: 43.56 | Achievement test: problem-solving exercises, multiple choice questions, true or false questions, open-ended questions | There were no differences between group | Aa: -0.04 (.02)  B: 0 (.01)  C: -0.07 (.03)  D: 0.05 (.05)  E: 0.05 (.04) |
| *Suárez‐Cunqueiro et al., 2017 | European Journal of Dental Education | Q2 | 109 | Spain | Jigsaw I | Dentistry | University | 12 hours (1 semester: 4X 3-h seminars) | NM | 31.2 | Achievement test  Perceptions of the Jigsaw approach five-point Likert-type questionnaire. | Jigsaw increased achievement. Students had positive Jigsaw opinion. | A: 0.21 (*.04*) |
| Study | Journal | SJR | *N total* | Geographic location | Jigsaw method | Discipline | Population | Duration | Age [y] | Percent of boys | Outcome measures | Results | ES *(var)* |
| *Tarhan & Sesen, 2012 | Chemistry Education Research and Practice | Q1 | 38 | Turkey | Jigsaw I | Chemistry | University | 1h15 | 18.5 | NM | Achievement tests: acid base theorie concept  Semi-structured interviews | The Jigsaw increased achievement.  Students had in general a positive opinion about Jigsaw. | A: 1.39 *(.13)* |
| *Tarhan et al., 2013 | Research in Science & Technological Education | Q2 | 61 | Turkey | Jigsaw I | Physics | Secondary school | 8 hours (2 weeks: 4 class/hours) | 12 | NM | Achievement tests: physical and chemical changes concept test  Semi-structured interviews | The Jigsaw increased achievement.  Student had positive Jigsaw opinion. | A: 2.57 *(.12)* |
| Theobald et al., 2017 | PLoS ONE | Q1 | 684 | United Stated | Jigsaw I | Biology | Secondary school | 5 hours | NM | 33.5 | Achievement test: student performance  Perceptions of student’s questionnaire | No significant result on achievement. Positive results on students’ interactions. | NA |
| *Ural et al., 2017 | Asia-Pacific Forum on Science Learning and Teaching | Q4 | 49 | Turkey | Jigsaw I | Sciences | Primary school | 6 hours (6 weeks) | Nd | NM | Achievement test  Motivation to learn and Attitude questionnaire | The Jigsaw increased achievement and motivation. | A: 0.01 *(.08)*  M: 0.6 *(.08)* |
| *Van Dat, 2016 | International Journal of Higher Education | Q4 | 80 | Vietnam | Jigsaw I | Management of Education and Administration | University | 18 hours (6 weeks) | 21.50 | 60.00 | Achievement tests: pre and post-test on MAE knowledge  Knowledge retention  Perceptions survey on generic teaching skills, student-centered skills, engagement with learning and powerful teacher. | Jigsaw increased achievement and retention. Jigsaw students appreciated most working with others and getting help, discussing and sharing information and teaching others, and enjoyed the jigsaw context. | A: 0.54 *(.05)* |
| Study | Journal | SJR | *N total* | Geographic location | Jigsaw method | Discipline | Population | Duration | Age [y] | Percent of boys | Outcome measures | Results | ES *(var)* |
| Van Dat & Lewis, 2012 | International Education Studies | Q3 | 80 | Vietnam | Jigsaw I | Math | University | 6 hours (6 weeks) | 21.53 | 60 | Achievement tests: MAE knowledge  Attitude | Jigsaw increased achievement and retention. Jigsaw students appreciated most working with others | NA |
| Walker & Crogan, 1998 | Journal of Community & Applied Social Psychology | Q1 | 103 | Australia | Jigsaw I | NM | Primary school | School A: 6 hours (4 weeks)  School B: 8 hours) | 11.5 | NM | Achievement test  Self-concept Scale (CSCS): Sociometric class survey: | Jigsaw produced significant improvements on measures of academic performance, liking of peers, and racial prejudice | NA |
| *Wilson, et al., 2017 | Currents in Pharmacy Teaching and Learning | Q1 | 94 | United States | Jigsaw I | Pharmaceutic | University | 12 hours (1 semester) | 23.6 | 38.3 | Achievement test  Students' perceptions | No statistical difference between groups on achievement. Students had positive perceptions on Jigsaw. | A: 0.46 *(.05)* |
| *Yapici, 2016 | Educational Research and Reviews | Q4 | 53 | Turkey | Jigsaw I | Sciences | Secondary school | 11 hours (11 weeks) | NM | NM | Science within time achievement test  Jigsaw Opinion Scale | The Jigsaw increased achievement.  Students had a positive opinion about the Jigsaw. | A: 2.65 *(.14)* |
| Study | Journal | SJR | *N total* | Geographic location | Jigsaw method | Discipline | Population | Duration | Age [y] | Percent of boys | Outcome measures | Results | ES *(var)* |
| Zacharia et al., 2011 | Educational Technology Research and Development | Q1 | 38 | Cyrius | Jigsaw I | Sciences | Secondary school | 9 hours | 14 | 52.63 | Screen and video date  Achievement test: conceptual test  Interviews | No significant difference on achievement and social relations. |  |
| Ziegler, 1981 | Human Organization | Q2 | 146 | Canada | Jigsaw II | NM | Secondary school | 8 hours (8 weeks) | Nd | 50 | Achievement test : curriculum test  Sociometric measure  Attitudinal questionnaire (measure to appreciation for social diversity) | Positive effects were obtained in all variables in favor of Jigsaw condition. | NA |

*Note*. SJR= Scimago Journal Rank; Q1 = Quartile 1; Q2 = Quartile 2; Q3 = Quartile 3; Q4 = Quartile 4; A = Achievement; M = Motivation; NA = No measured; NM = No mentioned R = Relatedness; ASE= Academic Self-esteem; TSE = Teaching Self-esteem.

**References**

*Abed, A. Z., Sameer, S. A., Kasim, M. A., & Othman, A. T. (2020). Predicting effect implementing the jigsaw strategy on the academic achievement of students in mathematics classes. *International Electronic Journal of Mathematics Education*, *15*(1), 1-7. <https://doi.org/10.29333/iejme/5940>

Abrami, P. C., Poulsen, C., & Chambers, B. (2004). Teacher motivation to implement an educational innovation: Factors differentiating users and non-users of cooperative learning. *Educational Psychology*, *24*(2), 201-216. <https://doi.org/10.1080/0144341032000160146>

*Akkus, A., & Doymuş, K. (2022). Effect of subject jigsaw and reading writing presentation techniques on academic achievement of 6th grade science students’ academic success in matter and heat unit. *Journal of Turkish Science Education*, *19*(2), 496-510.

Aronson, E., Blaney, N., Stephan, C., Sikes, J., & Snapp, M. (1978). *The Jigsaw classroom*. Beverly Hills, CA: Sage.

Aronson, E., & Patnoe, S. (2011). Cooperation in the Classroom: The Jigsaw Method (3rd ed.). London, England: Pinter & Martin.

*Arslan, A. (2016). Effect of Jigsaw I technique on teaching Turkish grammar. *Educational Research and Reviews, 11*(8), 635-641. <https://doi.org/10.5897/ERR2016.2709>

*Artut, P. D., & Tarim , K. (2007). The effectiveness of Jigsaw II on prospective elementary school teachers. *Asia‐Pacific Journal of Teacher Education, 35*(2), 129-141. <https://doi.org/10.1080/13598660701268551>

Azmin,N.H. (2015). Effect of the Jigsaw- Based Cooperative Learning Method on Student Performance in the General Certificate of Education Advanced-Level Psychology: An Exploratory Brunei Case Study. *International Education Studies, 9*(1), 91-106. <https://doi.org/10.5539/ies.v9n1p91>

*Aydin, A., & Biyikli, F. (2017). The Effect of Jigsaw Technique on the Students' Laboratory Material Recognition and Usage Skills in General Physics Laboratory-I Course. *Universal Journal of Educational Research, 5*(7), 1073-1082. <https://doi.org/10.13189/ujer.2017.050701>

Baken, E. K., Adams, D. C., & Rentz, M. S. (2022). Jigsaw method improves learning and retention for observation-based undergraduate biology laboratory activities. *Journal of Biological Education*, *56*(3), 317-322. <https://doi.org/10.1080/00219266.2020.1796757>

*Berger, R., & Hänze, M. (2009). Comparison of Two Small‐group Learning Methods in 12th‐grade Physics Classes Focusing on Intrinsic Motivation and Academic Performance. *International Journal of Science Education*, *31*(11), 1511-1527. <https://doi.org/10.1080/09500690802116289>

Berger, R., & Hänze, M. (2015). Impact of expert teaching quality on novice academic performance in the jigsaw cooperative learning method. *International Journal of Science Education*, *37*(2), 294-320. <https://doi.org/10.1080/09500693.2014.985757>

Blajvaz, B. K., Bogdanović, I. Z., Jovanović, T. S., Stanisavljević, J. D., & Pavkov-Hrvojević, M. V. (2022). The Jigsaw Technique in Lower Secondary Physics Education: Students’ Achievement, Metacognition and Motivation. *Journal of Baltic Science Education*, *21*(4), 545-557.

Blaney, N. T., Stephan, C., Rosenfield, D., Aronson, E., & Sikes, J. (1977). Interdependence in the classroom: A field study. *Journal of Educational Psychology, 69,* 121–128. <https://doi.org/10.1037/0022-0663.69.2.121>

Borenstein, M., Hedges, L. V., Higgins, J. P. T., & Rothstein, H. R. (2009). Introduction to meta-analysis. Chichester: John Wiley & Sons, Ltd.

Bratt, C. (2008). The Jigsaw classroom under test: no effect on intergroup relations evident. *Journal of Community & Applied Social Psychology*, *18*(5), 403–419.

Bridgeman, D. L. (1981). Enhanced role taking through cooperative interdependence: A field study. *Child Development*, *52*(4), 1231-1238.

Buchs, C., & Butera, F. (2009). Is a partner’s competence threatening during dyadic cooperative work? It depends on resource interdependence. *European Journal of Psychology of Education 24*, 145–154. <https://doi.org/10.1007/BF03173007>

Buchs, C., & Butera, F. (2015). Cooperative learning and social skills development. In Gillies R. M. (Ed.), Collaborative Learning: Developments in Research and Practice (pp. 201-217). New York: Nova Science.

Buchs, C., Butera, F., & Mugny, G. (2004a). Resource interdependence, student interactions and performance in cooperative learning. *Educational Psychology 24*, 291–314. <https://doi.org/10.1080/0144341042000211661>

Buchs, C., Pulfrey, C., Gabarrot, F., & Butera, F. (2010). Competitive conflict regulation and informational dependence in peer learning. *European Journal of Social Psychology 40*, 418–435. <https://doi.org/10.1002/ejsp.631>

Buchs, C., Filippou, D., Pulfrey, C., & Volpé, Y. (2017). Challenges for cooperative learning implementation: Reports from elementary school teachers. *Journal of Education for Teaching*, *43*(3), 296-306. <https://doi.org/10.1080/02607476.2017.1321673>

Buchs, C., Filippou, D., & Pulfrey, C. (2018). Reducing Threat in Cooperative Learning: The Role of Decentering. *International Review of Social Psychology, 31*(6), 1-7. Retrieved from <http://hdl.handle.net/20.500.12162/5520>

Casey, A., & Goodyear, V. (2015). Can cooperative learning achieve the four learning outcomes of physical education? A review of literature. *Quest, 67*(1), 56-72. <https://doi.org/10.1080/00336297.2014.984733>

Chang, W. L., & Benson, V. (2022). Jigsaw teaching method for collaboration on cloud platforms. *Innovations in Education and Teaching International*, *59*(1), 24-36. <https://doi.org/10.1080/14703297.2020.1792332>

Cheung, A. C., & Slavin, R. E. (2016). How methodological features affect affect sizes in education. *Educational Researcher, 45*(5), 283-292. <https://doi.org/10.3102/0013189X16656615>

Cochon Drouet, O., Lentillon-Kaestner, V., Roure, C., & Margas, N. (2022). The role of the type of sports on the effects of the Jigsaw method on students' motivation and moderate to vigorous physical activity in physical education. *Journal of Teaching of Physical Education,* *42*(2), 301-312. <https://doi.org/10.1123/jtpe.2021-0223>

Cochon Drouet, O., Fargier, F., Margas, N., & Lentillon-Kaestner, V. (2023). Self-reported Practice of Physical Education Teacher with the Jigsaw Method: Textual Analysis. *Educational Sciences, 13*(415), 1-14*.* [*https://doi.org/10.3390/ educsci13040415*](https://doi.org/10.3390/%20educsci13040415)

Cohen, E. (1994). *Designing Groupwork: Strategies for the Heterogeneous Classroom*. New York: Teachers College Press.

*Costouros, T. (2020). Jigsaw Cooperative Learning versus Traditional Lectures: Impact on Student Grades and Learning Experience. *Teaching & Learning Inquiry*, *8*(1), 154-172.

Crone, T. S., & Portillo, M. C. (2013). Jigsaw variations and attitudes about learning and the self in cognitive psychology. *Teaching of Psychology*, *40*(3), 246-251.

Deci, E. L., & Ryan, R. M. (2000). The" what" and" why" of goal pursuits: Human needs and the self-determination of behavior. *Psychological Inquiry, 11*(4), 227-268. <https://doi.org/10.1207/S15327965PLI1104_01>

Desforges, D. M., Lord, C. G., Ramsey, S. L., Mason, J. A., Van Leeuwen, M. D., West, S. C., & Lepper, M. R. (1991). Effects of structured cooperative contact on changing negative attitudes toward stigmatized social groups. *Journal of Personality and Social Psychology*, *60*(4), 531.

*Doymus, K. (2007). Effects of a cooperative learning strategy on teaching and learning phases of matter and one-component phase diagrams. *Journal of Chemical Education*, *84*(11), 1857-1860. <https://doi.org/10.1021/ed084p1857>

*Doymus, K. (2008a). Teaching chemical equilibrium with the jigsaw technique. *Research in science Education*, *38*(2), 249-260. <https://doi.org/10.1007/s11165-007-9047-8>

*Doymus, K. (2008b). Teaching chemical bonding through jigsaw cooperative learning. *Research in Science & Technological Education*, *26*(1), 47-57. <https://doi.org/10.1080/02635140701847470>

*Doymus, K., Karacop, A., & Simsek, U. (2010). Effects of jigsaw and animation techniques on students’ understanding of concepts and subjects in electrochemistry. *Educational Technology Research and Development*, *58*(6), 671-691. <https://doi.org/10.1007/s1142301091572>

Drouet, O., Millet, G., & Lentillon-Kaestner, V. (2020). Coopérer en éducation physique: Le Jigsaw, une méthode prometteuse? [Cooperate in physical education: the Jigsaw, a promising method?]. *eJRIEPS (eJournal de Recherche sur l'Intervention en Éducation Physique et en Sport), 46*, 21-50.

Dyson, B., & Casey, A. (2012). *Cooperative learning in physical education: A research-based approach*. (pp. 166-175). London, England: Routledge.

*Er, H. (2017). The Impact of Teaching the Subjects under" Science in Time" Unit in the Social Studies Class in the 7th Grade Using Jigsaw Technique on the Academic Success of the Students. *Universal Journal of Educational Research, 5*(5), 838-847. <https://doi.org/10.13189/ujer.2017.050516>

*Escalié, G., Legrain, P., & Lafont, L. (2018). L’apprentissage coopératif en «groupe d’experts» et la professionnalisation des futurs enseignants: un exemple en éducation physique et sportive [Cooperative learning in "expert groups" and the professionalization of future teachers: an example in physical education]. *Carrefours de l'Education, 46*, 161-176.

Fabes, R. A., Martin, C. L., & Hanish, L. D. (2018). Children and youth in a diverse world: Applied developmental perspectives on diversity and inclusion. *Journal of Applied Developmental Psychology*, *59*, 1–4. <https://doi.org/10.1016/j.appdev.2018.11.003>

Farmer, T. W., Hamm, J. V., Dawes, M., Barko-Alva, K., & Cross, J. R. (2019). Promoting inclusive communities in diverse classrooms: Teacher attunement and social dynamics management. *Educational Psychologist*, *54*(4), 286-305. <https://doi.org/10.1080/00461520.2019.1635020>

Fereday, J., & Muir-Cochrane, E. (2006). Demonstrating rigor using thematic analysis: A hybrid approach of inductive and deductive coding and theme development. *International Journal of Qualitative Methods*, *5*(1), 80-92. <https://doi.org/10.1177%2F160940690600500107>

Funder, D. C., & Ozer, D. J. (2019). Evaluating effect size in psychological research: Sense and nonsense. *Advances in Methods and Practices in Psychological Science, 2*(2), 156-168. <https://doi.org/10.1177/2515245919847202>

Gambari, A. I., & Yusuf, M. O. (2017). Relative effectiveness of computer-supported Jigsaw II, STAD and TAI cooperative learning strategies on performance, attitude, and retention of secondary school students in physics. *Journal of Peer Learning*, *10*(1), 76-94.

*Garcia, M. B. (2021). Cooperative learning in computer programming: A quasi-experimental evaluation of Jigsaw teaching strategy with novice programmers. *Education and Information Technologies*, *26*(4), 4839-4856.

*Ghaith, G., & El-Malak, M. A. (2004). Effect of Jigsaw II on literal and higher order EFL reading comprehension. *Educational Research and Evaluation*, *10*(2), 105-115. <https://doi.org/10.1076/edre.10.2.105.27906>

*Göçer, A. (2010). A comparative research on the effectivity of cooperative learning method and Jigsaw technique on teaching literary genres. *Educational Research and Reviews, 5*(8), 439-445.

Goh, J. X., Hall, J. A., & Rosenthal, R. (2016). Mini meta‐analysis of your own studies: Some arguments on why and a primer on how. *Social and Personality Psychology Compass*, *10*(10), 535-549. <https://doi.org/10.1111/spc3.12267>

*Gömleksi’z, M. N. (2007). Effectiveness of cooperative learning (Jigsaw II) method in teaching English as a foreign language to engineering students. *European Journal of Engineering Education*, *32*(5), 613-625. <https://doi.org/10.1080/03043790701433343>

Halimah, L., & Sukmayadi, V. (2019). The Role of "Jigsaw" Method in Enhancing Indonesian Prospective Teachers' Pedagogical Knowledge and Communication Skill. *International Journal of Instruction*, *12*(2), 289-304.

*Hamadneh, Q. M. S. (2017). The Effect of Using Jigsaw Strategy in Teaching Science on the Acquisition of Scientific Concepts among the Fourth Graders of Bani Kinana Directorate of Education. *Journal of Education and Practice, 8*(5), 127-134.

*Hänze, M., & Berger, R. (2007). Cooperative learning, motivational effects, and student characteristics: An experimental study comparing cooperative learning and direct instruction in 12th grade physics classes. *Learning and Instruction, 17*(1), 29-41. <https://doi.org/10.1016/j.learninstruc.2006.11.004>

Hattie, J. (2009). Visible learning: a synthesis of 800+ meta-analyses on achievement. London: Routledge Abingdon.

Hattie, J. (2017). 256 influences related to achievement. Visible Learning. <https://visible-learning.org/hattie-ranking-influences-effect-sizes-learning-achievement/>

Higgins, J. P., Thompson, S. G., Deeks, J. J., & Altman, D. G. (2003). Measuring inconsistency in meta-analyses. *Bmj*, *327*(7414), 557-560. <https://doi.org/10.1136/bmj.327.7414.557>

Holliday, D. C. (2000). *The Development of Jigsaw IV in a Secondary Social Studies Classroom*. Paper presented at the Midwest Educational Research Association (MWERA), Annual Conference, Chicago, IL.

*Hornby, G. (2009). The effectiveness of cooperative learning with trainee teachers*. Journal of Education for Teaching*, *35*(2), 161-168. <https://doi.org/10.1080/02607470902771045>

Johnson, D., & Johnson, R. (1989). *Cooperation and competition: Theory and research*. Edina: Interaction Book Company.

Johnson, D. W., & Johnson, R. T. (2002). Learning together and alone: Overview and meta‐analysis. *Asia Pacific Journal of Education*, *22*(1), 95-105. <https://doi.org/10.1080/0218879020220110>

Johnson, D. W., Johnson, R. T., & Smith, K. (2007). The state of cooperative learning in postsecondary and professional settings. *Educational Psychology Review, 19*(1), 15-29.

Johnson, D. W., Johnson, R. T., & Stanne, M. B. (2000). *Cooperative learning methods: A meta-analysis*. Minneapolis: University of Minnesota. Retrieved from <http://www.tablelearning.com/uploads/File/EXHIBIT-B.pdf>

Juvonen, J., Lessard, L. M., Rastogi, R., Schacter, H. L., & Smith, D. S. (2019). Promoting social inclusion in educational settings: Challenges and opportunities. *Educational Psychologist*, *54*(4), 250-270. <https://doi.org/10.1080/00461520.2019.1655645>

*Karacop, A., & Doymus, K. (2013). Effects of Jigsaw cooperative learning and animation techniques on students’ understanding of chemical bonding and their conceptions of the particulate nature of matter. *Journal of Science Education and Technology, 22*(2), 186-203. https://doi.org/10.1007/s10956- 012-9385-9

*Kilic, D. (2008). The effect of the Jigsaw technique on learning the concepts of the principles and methods of teaching. *World Applied Sciences Journal*, *4*(1), 109-114.

Kitayama, S., Markus, H. R., Matsumoto, H., & Norasakkunkit, V. (1997). Individual and collective processes in the construction of the self: self-enhancement in the United States and self-criticism in Japan. *Journal of Personality and Social Psychology, 72*(6), 1245. <https://psycnet.apa.org/doi/10.1037/0022-3514.72.6.1245>

*Koc, Y., Doymuş, K., Karaçöp, A., & Şimşek, Ü. (2010). The effects of two Cooperative learning strategies on the teaching and learning of the topics of chemical kinetics. *Journal of Turkish Science Education, 7*(2), 52-.65.

*Koç, Y., Yildiz, E., Çaliklar, S., & Simsek, U. (2016). Effect of Jigsaw II, reading-writing-presentation, and computer animations on the teaching of Light Unit. *Educational Research and Reviews*, *11*(20), 1906-1917

Kraft, M. A. (2020). Interpreting effect sizes of education interventions. *Educational Researcher*, *49*(4), 241-253. [https://doi.org/10.3102/0013189X20912798](https://doi.org/10.3102%2F0013189X20912798)

Kühberger, A., Fritz, A., & Scherndl, T. (2014). Publication bias in psychology: A diagnosis based on the correlation between effect size and sample Size. *Plos One, 9*(9), e105825. https://doi.org/10.1371/journal.pone.0105825

Kyndt, E., Raes, E., Lismont, B., Timmers, F., Cascallar, E., & Dochy, F. (2013). A meta-analysis of the effects of face-to-face cooperative learning. Do recent studies falsify or verify earlier findings? *Educational Research Review, 10*, 133-149. https://doi.org/10.1016/j.edurev.2013.02.002

Lazarowitz, R., Hertz‐Lazarowitz, R., & Baird, J. H. (1994). Learning science in a cooperative setting: Academic achievement and affective outcomes. *Journal of Research in Science Teaching*, *31*(10), 1121-1131.

Legrain, P., Escalié, G., Lafont, L., & Chaliès, S. (2019) Cooperative learning: a relevant instructional model for physical education preservice teacher training? *Physical Education and Sport Pedagogy*, *24*(1), 73-86. <https://doi.org/10.1080/17408989.2018.1561838>

Lentillon-Kaestner, V., & Patelli, G. (2017). Effet de l’alternance des formes de groupement sur le plaisir ressenti en éducation physique et sportive. *Staps, (2*), 61-74.

Lou, Y., Abrami, P. C., Spence, J. C., Poulsen, C., Chambers, B., & d’Apollonia, S. (1996). Within-class grouping: A meta-analysis. Review of Educational Research, 66(4), 423-458. <https://doi.org/10.3102/00346543066004423>

Lucker, G. W., Rosenfield, D., Sikes, J., & Aronson, E. (1976). Performance in the interdependent classroom: A field study. *American Educational Research Journal*, *13*(2), 115-123.

Maden, S. (2011). Effect of Jigsaw I Technique on Achievement in Written Expression Skill. *Educational Sciences: Theory and Practice*, *11*(2), 911-917.

Maison, T., Kurniawan, D. A., Sukarni, W., & Hoyi, R. (2021). Assessing Students' Attitudes towards Physics through the Application of Inquiry and Jigsaw Cooperative Learning Models in High Schools. *International Journal of Instruction*, *14*(4), 439-450.

Mari, J. S., & Gumel, S. A. (2015). Effects of jigsaw model of cooperative learning on self-efficacy and achievement in chemistry among concrete and formal reasoners in colleges of education in Nigeria. *International Journal of Information and Education Technology*, *5*(3), 196-199.

Maxwell, S. E. (2004). The persistence of underpowered studies in psychological research: Causes, consequences, and remedies. *Psychological Methods, 9*(2), 147-163. <https://doi/10.1037/1082-989X.9.2.147>

Moher, D., Liberati, A., Tetzlaff, J., Altman, D. G., & PRISMA Group*. (2009). Preferred reporting items for systematic reviews and meta-analyses: the PRISMA statement. *Annals of Internal Medicine*, 151(4), 264-269. <https://doi.org/10.7326/0003-4819-151-4-200908180-00135>

*Moreno, R. (2009). Constructing knowledge with an agent-based instructional program: A comparison of cooperative and individual meaning making. *Learning and Instruction, 19*(5), 433-444. <https://doi.org/10.1016/j.learninstruc.2009.02.018>

Moskowitz, J. M., Malvin, J. H., Schaeffer, G. A., & Schaps, E. (1983). Evaluation of a cooperative learning strategy. *American Educational Research Journal*, *20*(4), 687-696. [https://doi.org/10.3102/00028312020004687](https://doi.org/10.3102%2F00028312020004687)

Moskowitz, J. M., Malvin, J. H., Schaeffer, G. A., & Schaps, E. (1985). Evaluation of jigsaw, a cooperative learning technique. *Contemporary Educational Psychology*, *10*(2), 104-112. <https://doi.org/10.1016/0361-476X(85)90011-6>

Morge, L., & Toczek, M. C. (2009). L’expression des stéréotypes de sexe dans les situations d’entrée des séquences d’investigation en physique‑chimie. *Didaskalia*, *35*(1), 81-99.

Namaziandost, E., & Gilakjani, A. P. (2020). Enhancing pre-intermediate EFL learners’ reading comprehension through the use of Jigsaw technique. *Cogent Arts & Humanities*, *7*(1). <https://doi.org/10.1080/23311983.2020.1738833>

Oakes, D. J., Hegedus, E. M., Ollerenshaw, S. L., Drury, H., & Ritchie, H. E. (2019). U sing the J igsaw M ethod to T each A bdominal A natomy. *Anatomical sciences education*, *12*(3), 272-283. <https://doi.org/10.1002/ase.1802>

O’Leary, N., Barber, A., & Keane, H. (2019). Physical education undergraduate students’ perceptions of their learning using the jigsaw learning method. *European Physical Education Review*, *25*(3), 713-730. [https://doi.org/10.1177/1356336X18767302](https://doi.org/10.1177%2F1356336X18767302)

O’Leary, N., & Griggs, G. (2010). Researching the pieces of a puzzle: the use of a jigsaw learning approach in the delivery of undergraduate gymnastics. *Journal of Further and Higher Education*, *34*(1), 73-81. <https://doi.org/10.1080/03098770903477110>

O’Leary, N., Wattison, N., Edwards, T., & Bryan, K. (2015). Closing the theory–practice gap: physical education students’ use of Jigsaw learning in a secondary school. *European Physical Education Review, 21*(2), 176-194. <https://doi.org/10.1177/1356336X14555300>

ONU. (2015). The Agenda 2030: The Sustainable Development Goals. [L’Agenda 2030 : Les objectifs de développement durable]. Consulted in <https://www.un.org/sustainabledevelopment/fr/objectifs-de-developpement-durable>

Ovens, A., Dyson, B., & Smith, W. (2012) Implementing the cooperative learning model in physical education: the experience of New Zealand teachers. In: Dyson B and Casey A (eds) Cooperative Learning in Physical Education. A Research-Based Approach. London: Routledge, pp.15–26.

Oyserman, D., Coon, H. M., & Kemmelmeier, M. (2002). Rethinking individualism and collectivism: evaluation of theoretical assumptions and meta-analyses. *Psychological Bulletin*, *128*(1), 3-72. <https://psycnet.apa.org/doi/10.1037/0033-2909.128.1.3>

*Özdemir, E., & Arslan, A. (2016). The Effect of Self-regulated Jigsaw IV on University Students’ Academic Achievements and Attitudes towards English Course. *Journal of Education and Training Studies, 4*(5), 173-182. <https://doi.org/10.11114/jets.v4i5.1453>

Page, M. J., McKenzie, J. E., Bossuyt, P. M., Boutron, I., Hoffmann, T. C., Mulrow, C. D., Shamseer, L., Tetzlaff, J. M., Akl, E. A., Brennan, S. E., Chou, R., Glanville, J., Grimshaw, J. M., Hróbjartsson, A., Lalu, M. M., Li, T., Loder, E. W., Mayo-Wilson, E., McDonald, S., … Moher, D. (2021). The PRISMA 2020 statement: An updated guideline for reporting systematic reviews.  *The BMJ*, 372(71). <https://doi.org/10.1136/bmj.n71>

Patall, E. A. (2021). Implications of the open science era for educational psychology research syntheses. *Educational Psychologist*, 56(2), 142-160. <https://doi.org/10.1080/00461520.2021.1897009>

Peyrat, M. F. (2009). Tutoring and cooperative learning in junior high school. *Carrefours de l'Education*, *27*(1), 53-68.

Pianta, R. C., Belsky, J., Houts, R., & Morrison, F. (2007). Opportunities to learn in America's elementary classrooms. *Science*, *315*(5820), 1795-1796. <https://doi.org/10.1126/science.1139719>

Risko, V. J., Roller, C. M., Cummins, C., Bean, R. M., Collins, Block, Anders, C., et al. (2008). A critical analysis of research on reading teacher education. *Reading Research Quarterly,* 43, 252e288. <https://doi.org/10.1598.RRQ.43.3.3>

*Roseth, C. J., Lee, Y. K., & Saltarelli, W. A. (2019). Reconsidering Jigsaw social psychology: Longitudinal effects on social interdependence, sociocognitive conflict regulation, motivation, and achievement. *Journal of Educational Psychology*, *111*(1), 149. <https://doi.org/10.1037/edu0000257>

Sabourin, M. & Lehraus, K. (2008). Former des enseignants primaires à une approche coopé- rative: bilans et perspectives. In Y. Rouiller & K. Lehraus (Éds.), *Vers des apprentissages en coopération: rencontres et perspectives* (pp. 191-220). Berne: Peter Lang.

*Sahin, A. (2010). Effects of Jigsaw II technique on academic achievement and attitudes to written expression course. *Educational Research and Reviews, 5*(12), 777-787.

*Şahin, A. (2011). Effects of Jigsaw III technique on achievement in written expression. *Asia Pacific Education Review, 12*(3), 427-435. <https://doi.org/10.1007/s12564-010-9135-8>

*Sanaie, N., Vasli, P., Sedighi, L., & Sadeghi, B. (2019). Comparing the effect of lecture and Jigsaw teaching strategies on the nursing students' self-regulated learning and academic motivation: A quasi-experimental study. *Nurse Education Today*, *79*, 35-40. <https://doi.org/10.1016/j.nedt.2019.05.022>

*Santos Rego, M. A., & Moledo, M. D. M. L. (2005). Promoting interculturality in Spain: Assessing the use of the jigsaw classroom method. *Intercultural Education*, *16*(3), 293– 301. <https://doi.org/10.1080/14675980500212020>

*Sagsoz, O., Karatas, O., Turel, V., Yildiz, M., & Kaya, E. (2017). Effectiveness of Jigsaw learning compared to lecture‐based learning in dental education. *European Journal of Dental Education*, *21*(1), 28-32. <https://doi.org/10.1111/eje.12174>

Schäfer, T., & Schwarz, M. A. (2019). The meaningfulness of effect sizes in psychological research: Differences between sub-disciplines and the impact of potential biases. *Frontiers in Psychology*, *10*(813), 1-13. <https://doi.org/10.3389/fpsyg.2019.00813>

Scott, C. E., McTigue, E. M.; Miller, D. M. & Washburn, E. K. (2018). The what, when, and how of preservice teachers and literacy across the disciplines: A systematic literature review of nearly 50 years of research. *Teaching and Teacher Education,* 73, 1-13. <https://doi.org/10.1016/j.tate.2018.03.010>

*Shaaban, K. (2006). An initial study of the effects of cooperative learning on reading comprehension, vocabulary acquisition, and motivation to read. *Reading Psychology, 27*(5), 377-403. https://doi.org.10.1080/02702710600846613

Simmonds, M. Quantifying the risk of error when interpreting funnel plots. *Syst Rev* **4**, 24 (2015). <https://doi.org/10.1186/s13643-015-0004-8>

Slavin, R. E. (1986). Best-evidence synthesis: An alternative to meta-analytic and traditional reviews. *Educational Researcher, 15*(9), 5-11.

Slavin, R. E. (1990). Research on cooperative learning: Consensus and controversy. *Educational leadership*, *47*(4), 52-54.

Slavin, R. E. (2011). Instruction based on cooperative learning. In Mayer A. E. & Alexander P. A. (Eds.), *Handbook of Research on Learning and Instruction* (2nd edition, pp. 388-405). London: Routledge.

Slavin, R. E. (2015). Cooperative learning in elementary schools. *Education 3-13*, *43*(1), 5-14. <https://doi.org/10.1080/03004279.2015.963370>

*Souvignier, E., & Kronenberger, J. (2007). Cooperative learning in third graders’ Jigsaw groups for mathematics and science with and without questioning training. *British Journal of Educational Psychology, 77*(4), 755-771. <https://doi.org/10.1348/000709906X173297>

Stahl, R. J. (1994). *The Essential Elements of Cooperative Learning in the Classroom*. Bloomington, IN: Clearinghouse for Social Studies/Social Science Education ERIC Document Reproduction Service No. ED37088I). Retrieved November 2, 2004, from <https://www.ericfacifity.net/databases/ERIC_Digests/ed370881.html>

Stanczak, A. (2020). Is the "puzzle class" method effective in improving learning? [La méthode de la « classe puzzle » est-elle efficace pour améliorer l'apprentissage ?]. Doctoral dissertation, Clermont Auvergne University, France.

Stanczak, A., Darnon, C., Robert, A., Demolliens, M., Sanrey, C., Bressoux, P., Huguet, P., Buchs, C., Butera, F., & Consortium PROFAN. (2022). Do Jigsaw classrooms improve learning out- comes? Five experiments and an internal meta-analysis. *Journal of Educational Psychology,* 14(6), 1461–1476. <https://doi.org/10.1037/edu0000730>

Sterne, J. A., Sutton, A. J., Ioannidis, J. P., Terrin, N., Jones, D. R., Lau, J., ... & Higgins, J. P. (2011). Recommendations for examining and interpreting funnel plot asymmetry in meta-analyses of randomised controlled trials. *Bmj*, *343*. <https://doi.org/10.1136/bmj.d4002>

*Suárez‐Cunqueiro, M. M., Gándara‐Lorenzo, D., Mariño‐Pérez, R., Piñeiro‐Abalo, S., Pérez‐López, D., & Tomás, I. (2017). Cooperative learning in ‘Special Needs in Dentistry’for undergraduate students using the Jigsaw approach. *European Journal of Dental Education*, *21*(4), 64-71. <https://doi.org/10.1111/eje.12221>

Sutton, A. J., Song, F., Gilbody, S. M., & Abrams, K. R. (2000). Modelling publication bias in meta-analysis: a review. Statistical Methods in Medical Research, 9(5), 421–445. <https://doi.org/10.1177/096228020000900503>

*Tarhan, L., & Sesen, B. A. (2012). Jigsaw cooperative learning: acid-base theories. *Chemistry Education Research and Practice, 13*(3), 307-313. https://doi.org/10.1039/C2RP90004A

*Tarhan, L., Ayyıldız, Y., Ogunc, A., & Sesen, B. A. (2013). A Jigsaw cooperative learning application in elementary science and technology lessons: physical and chemical changes. *Research in Science & Technological Education, 31*(2), 184-203. <https://doi.org/10.1080/02635143.2013.811404>

Tatsioni, A., & Ioannidis, J.P.A. (2017). *Meta-Analysis*. In Quah, S.R (Ed.), International Encyclopedia of Public Health (Second Edition). Academic Press: Oxford, pp. 117-124. <https://doi.org/10.1016/B978-0-12-803678-5.00291-5>

Theobald, E. J., Eddy, S. L., Grunspan, D. Z., Wiggins, B. L., & Crowe, A. J. (2017). Student perception of group dynamics predicts individual performance: Comfort and equity matter*. PLoS ONE*, *12*(7), 1–16. <https://doi.org/10.1371/journal.pone.0181336>

Thornton, A., & Lee, P. (2000). Publication bias in meta-analysis. *Journal of Clinical Epidemiology, 53*(2), 207–216. <https://doi.org/10.1016/S0895-4356(99)00161-4>

Torgerson, C., Porthouse, J., & Brooks, G. (2005). A systematic review of controlled trials evaluating interventions in adult literacy and numeracy. *Journal of Research in Reading*, 28, 87e107.

UNESCO (2009). *Principes directeurs pour l’inclusion dans l’éducation*. Paris, France : Organisation des Nations Unies pour l’éducation, la science et la culture.

*Ural, E., Ercan, O., & Gençoğlan, D. M. (2017). The effect of Jigsaw technique on 6th graders' learning of force and motion unit and their science attitudes and motivation. *Asia-Pacific Forum on Science Learning & Teaching*, *18*(1), 1-21.

Uttl, B.; White, C. A.; Wong Glez, D. (2017). Meta-analysis of faculty´s teaching effectiveness: Student evaluation of teaching ratings and student learning are not related. *Studies in Educational Evaluation*, 54, 22-42. <https://doi.org/10.1016/j.stueduc.2016.08.007>

Valentine, J. C., Pigott, T. D., & Rothstein, H. R. (2010). How many studies do you need? A primer on statistical power for meta-analysis. *Journal of Educational and Behavioral Statistics*, *35*(2), 215-247. https://doi.org/ 10.3102/1076998609346961

*Van Dat, T. (2016). The Effects of Jigsaw Learning on Students' Knowledge Retention in Vietnamese Higher Education. *International Journal of Higher Education*, *5*(2), 236-253.

Van Dat, T., & Lewis, R. (2012). Effects of Cooperative Learning on Students at An Giang University in Vietnam. *International Education Studies*, *5*(1), 86-99. <http://dx.doi.org/10.5430/ijhe.v1n2p9>

Velàzquez, C. (2012a). Putting cooperative learning and physical activity into practice with primary students. In: Dyson B and Casey A (eds) Cooperative Learning in Physical Education. A Research-Based Approach. London: Routledge, pp.59–74.

Veroniki, A. A., Jackson, D., Viechtbauer, W., Bender, R., Bowden, J., Knapp, G., ... & Salanti, G. (2016). Methods to estimate the between‐study variance and its uncertainty in meta‐analysis. *Research Synthesis Methods*, 7(1), 55-79. <https://doi.org/10.1002/jrsm.1164>

Vives, E. (2021). Mécanismes cognitifs et psycho‐sociaux impliqués dans l’apprentissage coopératif Jigsaw : études expérimentales en milieu scolaire. Doctoral Thesis, University of Aix-en-Provence, France.

Voyles, E. C., Bailey, S. F., & Durik, A. M. (2015). New pieces of the jigsaw classroom: increasing accountability to reduce social loafing in student group projects. *The New School Psychology Bulletin, 13*(1), 11-20.

Walker, I., & Crogan, M. (1998). Academic performance, prejudice, and the jigsaw classroom: New pieces to the puzzle. *Journal of Community & Applied Social Psychology*, *8*(6), 381-393. [https://doi.org/10.1002/(SICI)1099-1298(199811/12)8:6<381::AID-CASP457>3.0.CO;2-6](https://doi.org/10.1002/(SICI)1099-1298(199811/12)8:6%3c381::AID-CASP457%3e3.0.CO;2-6)

Williams, D. (2004). Improving race relations in higher education: The jigsaw classroom as a missing piece to the puzzle. *Urban education*, *39*(3), 316-344. <https://doi.org/10.1177/0042085904263063>

*Wilson, J. A., Pegram, A. H., Battise, D. M., & Robinson, A. M. (2017). Traditional lecture versus jigsaw learning method for teaching medication therapy management (MTM) core elements. *Currents in Pharmacy Teaching and Learning*, *9*(6), 1151-1159.

*Yapici, H. (2016). Use of Jigsaw technique to teach the unit" science within time" in secondary 7th grade social sciences course and students' views on this technique. *Educational Research and Reviews, 11*(8), 773. <https://doi.org/10.1177/0042085904263063>

Zacharia, Z. C., Xenofontos, N. A., & Manoli, C. C. (2011). The effect of two different cooperative approaches on students’ learning and practices within the context of a WebQuest science investigation. *Educational Technology Research and Development*, *59*(3), 399–424. <https://doi.org/10.1007/s11423-010-9181-2>

Ziegler, S. (1981). The effectiveness of cooperative learning teams for increasing cross-ethnic friendship: Additional evidence. *Human Organization*, *40*(3), 264-268.
